# Supported Ce/Zr pyrochlore monolayers as a route to single cerium atom catalysts with low temperature reducibility

**DOI:** 10.1016/j.isci.2023.107506

**Published:** 2023-07-28

**Authors:** Jose M. Montes-Monroy, Ramón Manzorro, Lidia E. Chinchilla, William E. Celín, Jose J. Calvino, Jose A. Pérez-Omil

**Affiliations:** 1 Departamento de Ciencia de los Materiales e Ingeniería Metalúrgica y Química Inorgánica, Facultad de Ciencias, Universidad de Cádiz, 11510 Puerto Real, Spain; 2 Instituto de Microscopía Electrónica y Materiales (IMEYMAT), Facultad de Ciencias, Universidad de Cádiz, 11510 Puerto Real, Spain

**Keywords:** Chemistry, Catalysis, Materials Chemistry

## Abstract

The combination of structural characterization at atomic resolution, chemical data, and theoretical insights has revealed the unique nanostructures which develop in ceria supported on yttria-stabilized zirconia (YSZ) after being submitted to high-temperature reducing treatments. The results show that just a small ceria loading is needed for creating a supported Zr-rich pyrochlore (111) nanostructure, resembling the structure of single cerium atom catalysts. The specific atomic arrangement of this nanostructure allows to explain the improvement of the reducibility at low temperature. The reduction mechanism can be extrapolated to ceria-zirconia mixed oxides with pyrochlore-like cationic ordering, exposing Zr-rich (111) surfaces. The results gathered here provide key information to understand the redox behavior of these types of systems, which may contribute to improving the design of new ceria-zirconia based materials, with lower content of the lanthanide element, nearly 100% cerium atom utilization, and applications in environmental catalysis.

## Highlights

• YSZ-supported Zr-rich pyrochlore (111) monolayer prepared with minimum amount of Ce
• This strategy leads to single cerium atom arrangement with the highest cerium density
• Subsurface oxygen movement near isolated Ce atoms is key to understand reducibility
• Subsurface oxygen is also key in other systems with Zr-rich pyrochlore (111) surfaces

## Graphical abstract

**Figure:**
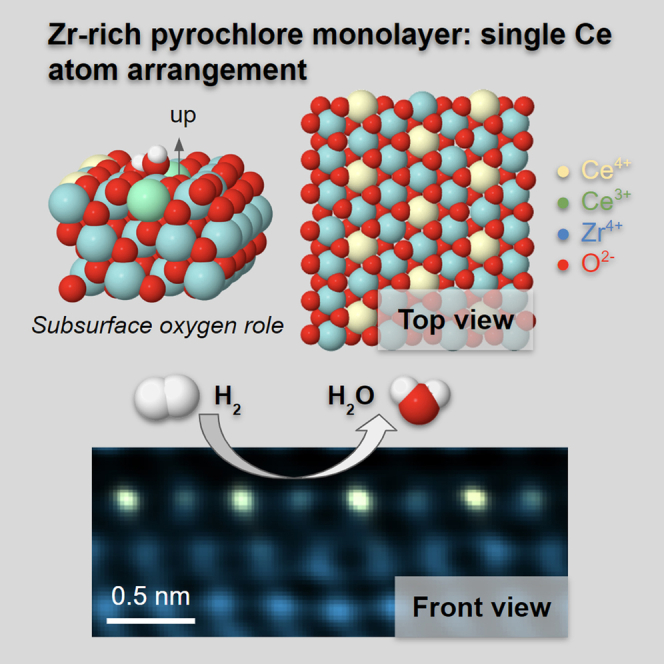

## Introduction

Ceria has been widely investigated, both experimentally and theoretically, as a support for catalysts due to its singular chemical and redox properties. During the last decades large efforts have been devoted to rationalizing their functionality and improving their performance.^1^^,^^2^^,^^3^^,^^4^^,^^5^^,^^6^^,^^7^^,^^8^^,^^9^^,^^10^

The increasing pressure on the utilization of critical raw materials has more recently raised interest in the development of new catalyst formulations with lower content of lanthanide elements.^11^ Single atom catalysts (SACs) have drawn great attention in this context, due to their potential to optimize the usage efficiency by maximizing the dispersion of the active phase. SACs in a variety of nanostructures have proven to exhibit high catalytic activity and selectivity in different processes, which allows us to consider them a bridge between heterogeneous and homogeneous catalysis.^12^^,^^13^^,^^14^^,^^15^ Recently, several works have reported an exceptional catalytic performance of single cerium atom catalysts in different redox reactions.^16^^,^^17^

Preparing supported catalysts has been the traditional way to decrease the content of lanthanides in the formulation of the catalyst to values in the order of 15% CeO_2_. Zirconia- and yttria-stabilized zirconia (YSZ)-supported ceria systems have been recently investigated.^18^^,^^19^^,^^20^ Temperature-programmed reduction treatments have revealed that the reducibility of YSZ-supported ceria can be largely enhanced after a so-called strong reduction mild oxidation (SRMO) treatment, which consists in a reduction at 900°C in a flux of H_2_ (5% in Ar) for several hours followed by an oxidation at 500°C in O_2_ (5% in He). The redox behavior of this SRMO-treated YSZ-supported catalyst is also superior to that of the bulk Ce/Zr mixed oxide of the same composition submitted to the same treatment. By analogy with the behavior observed in bulk ceria-zirconia materials, this was explained by the formation of a few-layers thick oxidized pyrochlore structure coherently grown onto (111) YSZ surfaces.^18^^,^^21^ Thus, atomically resolved chemical images recorded by energy dispersive X-ray spectroscopy (XEDS) confirmed the presence of cerium atoms in the top-most (111) planes of the YSZ crystals after the SRMO treatment, though it was not possible to evidence the ordering of the cations characteristic of the pyrochlore phase. In any case, these data definitely showed that 2D supported nanostructures provide an efficient route to decrease the content of the active component in the formulation of catalysts without deteriorating its redox performance.^18^

In this scenario, the first objective of this work has been to explore the possibility to further reduce the ceria loading in the CeO_2_/YSZ system, maximizing the cerium atom utilization, without deteriorating or even improving functionality, which is the ultimate goal of single atom catalysis. In the particular case of ceria, this is very closely connected to the redox response at low temperature. To this end, samples with a ceria loading equal or inferior to one monolayer were prepared. Aberration(Cs)-corrected scanning/transmission electron microscopy was used to characterize at atomic level the surface nanostructures present in the samples, both as prepared and submitted to SRMO treatments. This information was key to establish a correlation between nanostructure and redox response.

In relation to the synthetic goals, CO oxidation studies on Au/YSZ-supported ceria have evidenced that prolonged exposure to reaction conditions, which can be considered slightly oxidizing, spreads 3D-type cerium oxide nanoparticles into pure (111) ceria layers coherently grown on top of the YSZ (111) planes.^19^ It has been also reported that the ceria phase remains on the surface of YSZ during oxidation treatments under flowing O_2_ (5% in He) at temperatures up to 900°C (severe oxidation, or SO treatments).^21^ Taking this into account, the samples were submitted to an oxidizing pretreatment, prior to the SRMO cycle, in order to guarantee the complete dispersion of the ceria phase. With all this information in mind, it seems that the application of a preparation protocol, to very low-loading CeO_2_/YSZ catalyst, which includes a first SO treatment, followed by an SRMO one, could open a route to synthesize Ce/Zr pyrochlore-like monolayers supported on YSZ crystallites.

The second objective of this contribution has been to achieve a fundamental understanding about the formation of pyrochlore nanostructures in the YSZ-supported ceria system and clarify why these specific nanostructures are able to lower the reduction temperature under hydrogen. In this respect, density functional theory (DFT) has proven as an excellent tool to understand both the reduction process and the interaction of hydrogen with both bulk CeO_2_ and Ce/Zr pyrochlore structures.^22^^,^^23^^,^^24^^,^^25^^,^^26^^,^^27^^,^^28^^,^^29^^,^^30^^,^^31^^,^^32^ Therefore, an in-depth DFT study has been performed starting from structural models inspired from the experimental Cs-corrected scanning/transmission electron microscopy data. Special attention has been paid to elucidate the possible role of the reduction mechanism under hydrogen of the oxygen layer lying just underneath the top-most cation layer and the particular spatial distribution of the cerium atoms on the surface.

Finally, as a third objective, this work also pursues establishing a connection between bulk Ce/Zr mixed oxides and CeO_2_/YSZ system with different ceria loadings. To make this issue more specific, the following previous findings have to be considered:

(a) To prepare single-phase polycrystalline Ce/Zr pyrochlore oxide (a so-called kappa phase, ***κ***-CeZrO_4_), prolonged reduction treatments at very high temperature (>1200°C) are required.^33^^,^^34^^,^^35^ Theoretical studies have proven key to understanding the stability and reduction behavior of these pyrochlore structures.^29^^,^^30^^,^^31^^,^^32^^,^^36^^,^^37^^,^^38^^,^^39^^,^^40^^,^^41^
(b) High-resolution (scanning) transmission electron microscopy (HR-S/TEM) data obtained from bulk Ce/Zr mixed oxide systems submitted to SRMO treatments have evidenced the formation of nuclei of oxidized pyrochlore-like structure.^42^^,^^43^^,^^44^^,^^45^ Likewise, a correlation has been established between the pyrochlore nuclei and the enhancement of the mixed oxide reducibility at low temperature.^45^^,^^46^^,^^47^^,^^48^^,^^49^
(c) Electron tomography and HR-S/TEM experiments have also evidenced an increase of the percentage of exposed {111} facets in bulk ceria-zirconia crystallites containing pyrochlore nuclei with a preferential termination in Zr-rich planes.^44^^,^^46^^,^^50^ It is worth mentioning at this point that due to the alternation of Ce-rich (111) planes (with a 75/25 Ce/Zr molar ratio) and Zr-rich planes (25/75 M ratio) in the bulk pyrochlore structure, the composition of their top-most surface cationic plane could be either Ce rich or Zr rich.

Therefore, the third objective of this work can be reformulated as investigating the relationship, from the point of view of structure and chemical properties, between the (111) surface of the pyrochlore nuclei in bulk Ce/Zr mixed oxides, the pyrochlore (111) nanostructures in the CeO_2_/YSZ system, and the eventual formation of YSZ-supported Zr-rich pyrochlore (111) monolayers.

All the objectives have been addressed with both an experimental and a theoretical approach. The combination of atomic resolution data and DFT calculations has allowed to unveil fundamental aspects of the singular reduction mechanism of YSZ-supported ceria-based nanostructures and the existence of atomically dispersed cerium species on the sample. This information can be used to improve the design of materials based on the ceria-zirconia system for particular applications, specially in the field of environmental catalysis.

## Results and discussion

### Experimental characterization: High-resolution scanning transmission electron microscopy (HR-STEM) data and H_2_-temperature programmed reduction (TPR) experiments

Two samples have been prepared and characterized to obtain data which complement other results already reported elsewhere.^18^^,^^19^^,^^21^ Sample CZ1 consists in a YSZ support of middle specific surface area onto which an amount of ceria corresponding to less than one monolayer (0.71) has been supported. Figure S1 shows a representative high resolution transmission electron microscopy (HRTEM) image of this sample where a 3D ceria nanoparticle grown on top of the support surface can be clearly observed. HAADF images of this sample and XEDS elemental maps illustrate the dispersion state of ceria on the support. In good agreement with previous literature,^21^ ceria concentrates in the form of particles in the 5–20 nm size range distributed along the surface of the support crystallites. Of course, the formation of small clusters or atomic chains cannot be disregarded.

**Figure 1:**
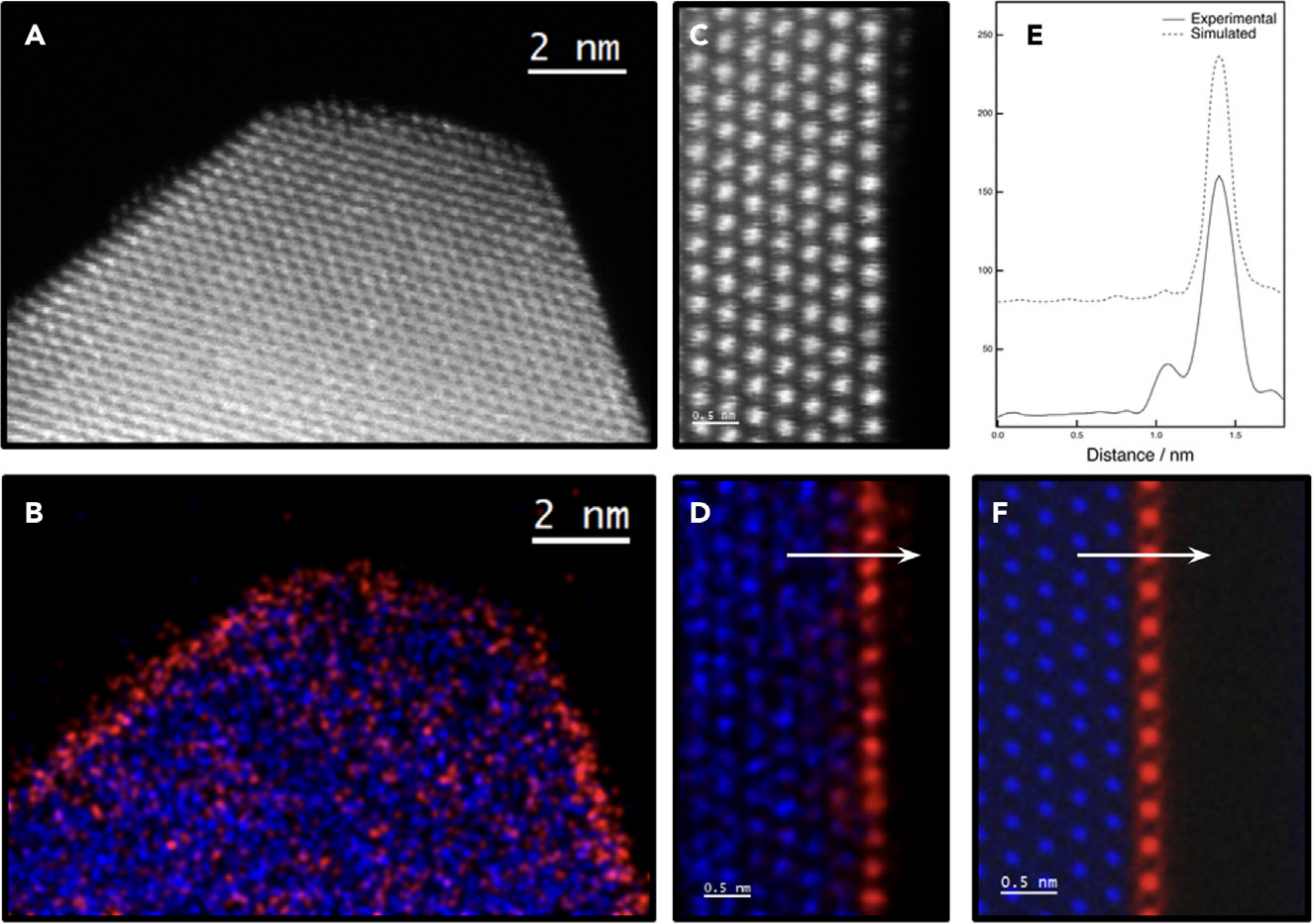
STEM results corresponding to the CZ1 sample after an SO treatment (A) Low-magnification HAADF STEM image showing a representative nanostructure. (B) XEDS elemental map acquired over the region displayed in the HAADF image. (C) Atomic-resolution HAADF image showing a representative surface of the system. (D) XEDS elemental map corresponding to the high-resolution HAADF image. (E) Intensity profile along a direction perpendicular to the surface on the experimental and simulated Ce maps. (F) XEDS map calculated with the muSTEM code. Ce and Zr spatial distribution are displayed in red and blue colors, respectively.

Figures 1A and 1B show a low-magnification high-angle annular dark-field (HAADF) image and an XEDS elemental map recorded on CZ1 after an SO treatment. 2D nanostructures on top of YSZ are detected which indicate an increase in the dispersion of ceria phase after this treatment. Figure 1C and 1D show an atomically resolved HAADF image and the corresponding high-resolution XEDS elemental map representative of the {111} surfaces, which are the most exposed faces in this support. Note that the ceria (111) monolayer grows in a perfect parallel epitaxy on top of the YSZ (111) surface (both surface layer and support are fluorite-like phases, Figure S2). This structural relationship agrees with the tendency of ceria to wet the (111) surfaces of YSZ under oxidizing conditions, in good agreement with DFT calculations shown in the following section.

A careful quantitative analysis of the area in image 1D reveals a Ce/Zr ratio close to 58/43 in the first layer, i.e., below the nominal ceria coverage of 0.71 monolayers. HAADF-STEM intensity profiles, Figure S2, indicate that Ce and Zr do not become ordered within the surface layer but, instead, remain disordered in the form of a solid-solution, fluorite-type oxide monolayer. The second layer, just beneath, presents a Ce/Zr ratio close to 14/86 (Figure 1E). A more precise quantitative analysis of these XEDS maps requires the use of simulations taking into account the eventual influence of crosstalk effects between adjacent atomic columns. Therefore, calculations were performed considering the quantum excitation of phonons (QEP) theory, using the ***μ***STEM software,^51^ starting from a model containing a supported ceria monolayer (100% CeO_2_) (Figure 1F). The complete set of simulations, Figure S3, indicates that, despite Ce atoms locating only in the first layer of the model, a small amount (≃ 4% mol.) of Ce is detected on top of the Zr columns of the second layer, due to channeling effects, for a model thickness of 40 nm. If this effect is taken into account to correct the quantitative data commented above, the amount of Ce in the first and second layers would be closer to 62% and 10%, respectively. Therefore, Ce-Zr mixing within both the first and the second layers has to be admitted and the supported ceria layer formed after an SO treatment of the CZ1 sample is not made of pure CeO_2_ but, instead, of a fluorite-like Ce-rich (62%) Ce/Zr mixed oxide.

It is worth mentioning at this point that bulk pyrochlore-like Ce/Zr mixed oxides decompose into two fluorite Ce/Zr mixed oxide solid solutions of different compositions when submitted to an oxidation treatment at high temperature: a Ce-rich phase containing approximately 30% ZrO_2_ and a second phase, Zr-rich, with approx. 14% CeO_2_ content, as reported elsewhere.^52^ Note that these compositions are related with those measured in the first and second layers in the CZ1 sample. This could explain the chemical stability of these two layers.

From a structural point of view, the detailed analysis of the image in Figure 1C evidences both a shrinkage of the ceria lattice parameter, to allow for a perfect match with the YSZ structure, and an outward shift (around 0.22 Å) in the direction perpendicular to the surface of the top-most atomic plane (that corresponding to the Ce-rich monolayer) with respect to the YSZ (111) planes (see Figure S2C), in good agreement with previous reported results^19^ and the calculations presented in the next theoretical section of this work.

**Figure 2:**
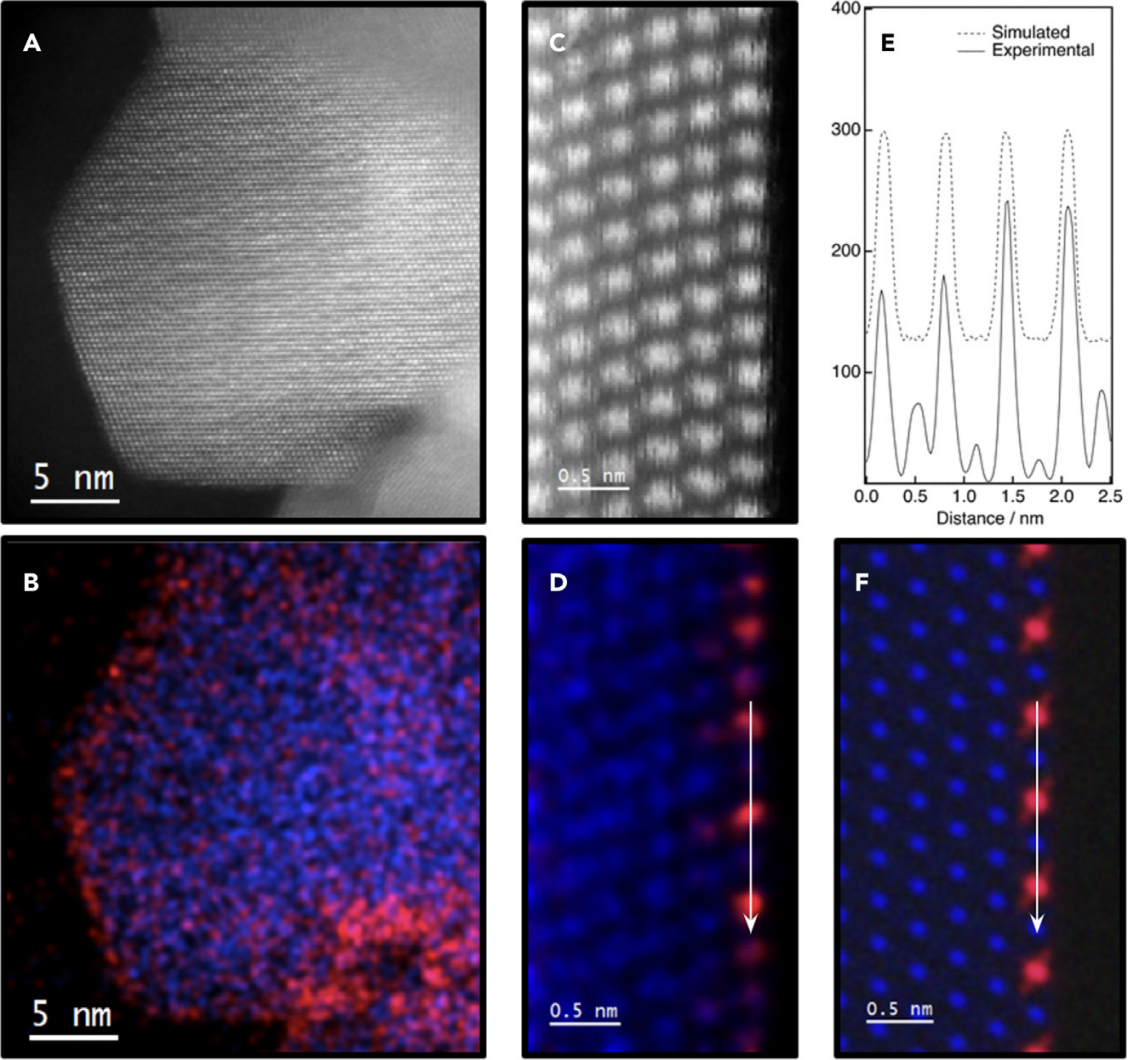
STEM results corresponding to the CZ1 sample after an SO-SRMO treatment (A) Low-magnification HAADF STEM image. (B) The corresponding low-magnification XEDS elemental map, showing (in red) Ce highly dispersed all over the surface. (C) Atomic-resolution HAADF STEM image. (D) XEDS elemental map corresponding to the region observed in the high-resolution HAADF STEM image. (E) Intensity profiles recorded along the top-most cationic plane (parallel to the surface) over the experimental and simulated Ce XEDS maps, which reveal the occurrence of the cationic order characteristic of the pyrochlore phase. (F) Ce and Zr XEDS simulated maps obtained through the ***μ***STEM code. In all the elemental maps Ce and Zr are shown in red and blue, respectively.

Figures 2A and 2B show representative results of the STEM study of the CZ1 sample after an SO-SRMO treatment. The images account again for the dispersion state of the ceria phase on top of the support. Note that in this case some local inhomogeneities in the spatial distribution of the ceria phase are observed (Figure S4). The atomically resolved HAADF and XEDS maps gathered in Figures 2C and 2D evidence more detailed aspects of the structure of this sample. Once more, the ceria phase seems to concentrate just in the first, top-most surface layer with only a minor contribution in the second one, being the (111) surfaces the most representative. The intensity profile taken along the direction of the first layer in the XEDS map, Figure 2E, clearly evidences patches of a cationic ordering characteristic of the pyrochlore phase, with an alternation of Ce-rich and Zr-rich columns. By selecting an integration window which contains an even number of cation columns, it was possible to estimate, in a first approximation, the chemical composition of this layer. In particular, a value close to 25/75 was obtained for the Ce/Zr molar ratio. Clearly this composition is below that expected for the nominal at % Ce deposited during the preparation of CZ1 sample (corresponding to 0.71 monolayers), even if a small fraction of Ce would eventually become mixed with Zr in the second subsurface cation layer, as observed in the SO sample. The alternating chemical composition of neighboring atomic columns, one containing just Zr (i.e., with a negligible amount of Ce) followed by one with a Ce content around 50%, with an averaged Ce/Zr ratio close to 25/75 on the plane, clearly evidences the pyrochlore-like cationic ordering, characteristic of a Zr-rich (111) plane in bulk Ce_2_Zr_2_O_7_ observed along [110] direction.^53^ The remaining Ce content is very likely involved in the ceria accumulations detected in the low-magnification images.

Due to the alternation of Zr-rich and Ce-rich (111) planes in the bulk pyrochlore structure, it is possible to create a monolayer enriched in one of these two elements, the Zr-rich monolayer being the one with 25% Ce, where all the Ce atoms are surrounded by Zr ones, resembling a cerium SAC arrangement with the highest density of isolated cerium atoms. However, a simple visual inspection of the XEDS map suggests that the top-most surface of the YSZ crystal corresponds to a Ce-rich plane, with a small amount of Zr. This apparent contradiction with the quantitative XEDS analysis can be explained through simulations. Thus, Figure 2F shows an XEDS elemental map calculated using the ***μ***STEM code, which takes into account the channeling effects using the QEP algorithm, for a single supported pyrochlore Zr-rich monolayer, with a 25/75 Ce/Zr molar ratio. Note that, also in this case, despite the much lower Ce content in the surface layer of the model (25%), the visual inspection of the simulated image suggests a Ce-rich monolayer. This effect is due to the fact that the cross-section of the L-signal for Ce is much larger than that of the Zr K-signal. Therefore, care must be taken when interpreting the relative chemical composition of atomic layers of these two elements on the basis of a simple visual inspection.

**Figure 3:**
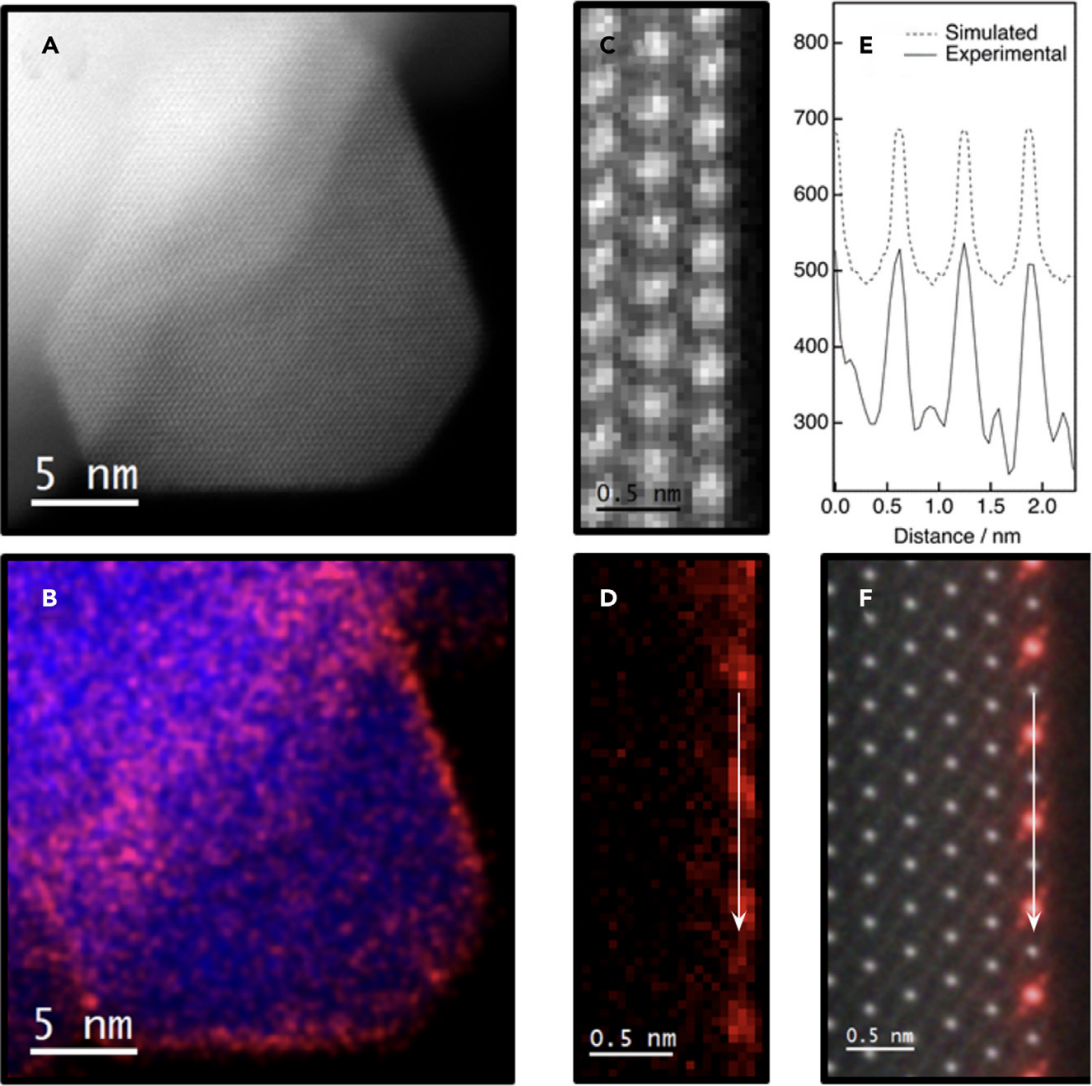
STEM results corresponding to the CZ2 sample after an SO-SRMO treatment (A) Representative low magnification HAADF STEM image. (B) Low-magnification XEDS elemental map from the HAADF image. (C) Atomic-resolution HAADF image from a representative surface. (D) Ce-M_4,5_ EELS signal acquired simultaneously to the HAADF image, illustrating the characteristic pyrochlore cationic order. (E) Experimental and simulated Ce intensity profiles acquired over the top-most cationic plane. (F) Simulated Ce-M_4,5_ EELS atomic resolution map together with the simulated HAADF STEM image, both calculated using the muSTEM code. Ce and Zr are represented, respectively, in red and blue.

The STEM results obtained from sample CZ1 evidence that, after an SRMO treatment, ceria wets the surface of the support as a Zr-rich (25% Ce mol.) pyrochlore type monolayer, despite the Ce loading largely exceeding that corresponding to a full coverage of the support surface with such a structure. Therefore, a second sample called CZ2 was prepared depositing the amount of ceria corresponding to just 0.25 monolayers. This sample was submitted to the SO-SRMO treatment (Figure S5), showing a homogeneous dispersion of the ceria phase. Figure 3 gathers structural (HAADF images) and compositional information (electron energy loss spectroscopy [EELS] maps) of this sample at both low (A-B) and high magnification (C-D) scales. Note that the EELS signal, Figure 3D, depicts the periodic intensity change expected for the cationic ordering characteristic of the pyrochlore phase. The EELS-simulated image, calculated using the ***μ***STEM code, shown in Figure 3E, confirms in fact the formation of a Zr-rich pyrochlore type monolayer.

In small CZ2 crystals after the SO-SRMO treatment, like the one shown in Figure S6, it is possible to detect some modulations in top-view HAADF images. If an intensity profile is recorded on selected areas of these HAADF images, along the direction perpendicular to the (111) planes (Figures S6C and S6D), peaks of high and slightly lower intensity are observed to alternate. In order to interpret this general feature, a model was built in which a faceted (111) YSZ crystallite, covered by a Zr-rich pyrochlore monolayer, is viewed along the [110] direction (Figure S7A). A detailed comparison of the contrasts observed in the simulations corresponding to this model, with and without the Zr-rich pyrochlore monolayer, indicates that it is possible to distinguish both situations, Figures S7B and S7C. In fact, the alternating higher/lower intensity pattern characteristic of the experimental images is only observed in the simulation obtained for the model in which the pyrochlore-type monolayer is considered. Therefore, these simulations provide further evidence of the existence of a Zr-rich pyrochlore monolayer covering the surface of CZ2 sample after an SO-SRMO treatment.

Besides this, only an isolated ceria-phase accumulation in the form of a pyrochlore-like nanocrystal exposing (111) surfaces (Figure S8) was found in the whole set of images recorded on the CZ2 sample after an SRMO treatment. This fact evidences an excellent dispersion of the ceria phase in the CZ2 sample, compared with CZ1. In conclusion, these results show, therefore, that it is possible to create a monolayer of the supported Zr-rich pyrochlore by decreasing the loading of ceria to just that corresponding to 0.25 monolayer.

**Figure 4:**
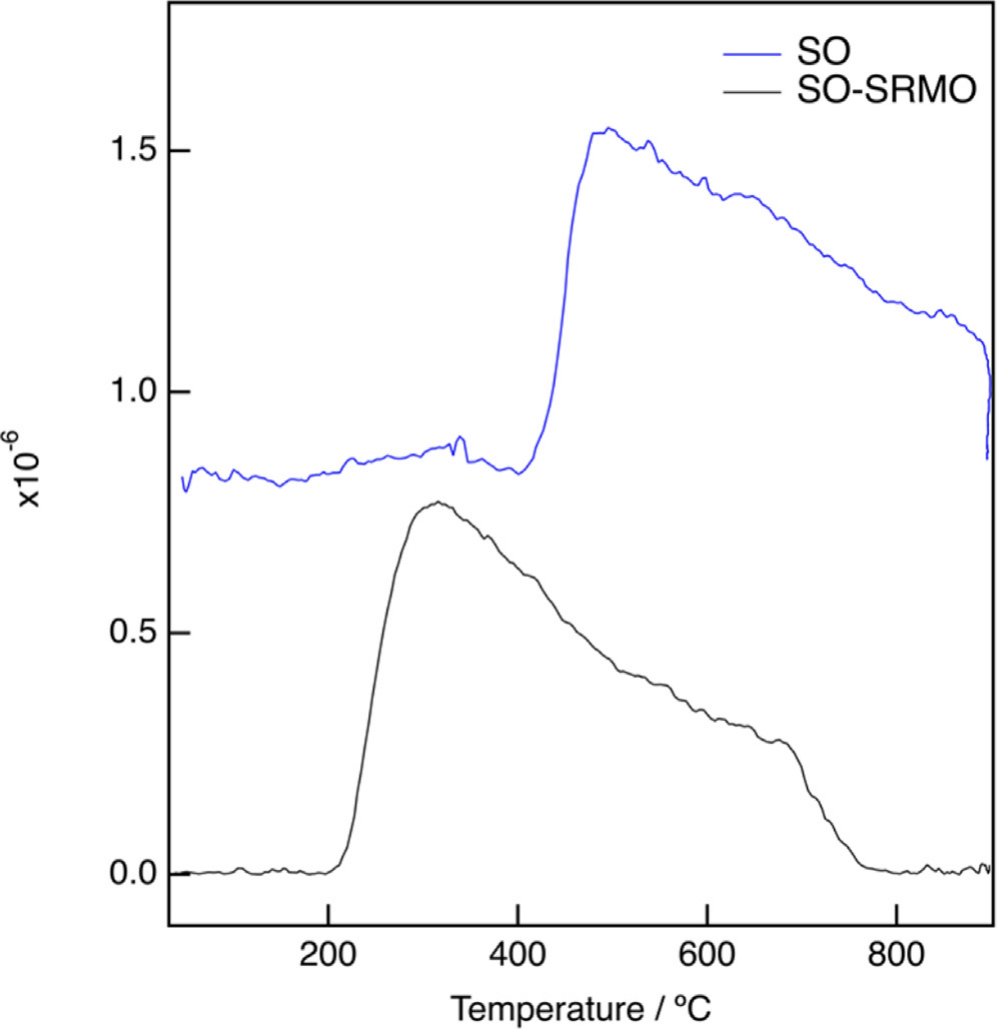
H_2_-TPR (m/c 18) experiments corresponding to sample CZ2 submitted to a thermal pretreatment After an SO redox cycle, plot in blue at the top, and after an SO-SRMO treatment, plot in black at the bottom.

To determine the effect of decreasing the total Ce content to just a quarter of a monolayer on the redox performance of the system, H_2_-TPR experiments using a mass spectrometer (MS) were performed after the SO and SO-SRMO treatments (Figure 4). Note that the onset of reduction shifts downwards by more than 200°C, after the SRMO cycle, which demonstrates the improvement in the reducibility of this sample after this special type of treatment. DFT calculations gathered in the next section prove that the formation of just one pyrochlore monolayer could be behind the enhancement of the reducibility at low temperature.

These later results are quite relevant for the formulation of new catalysts based on CeO_2_/YSZ since they point out to the possibility of decreasing further, below one monolayer, the general content of Ce while maintaining the redox performance.

The corresponding TPR experiments using a thermal conductivity detector (TCD) are shown in Figure S9. In both cases, a hydrogen release at low temperature can be observed due to desorption of hydrogen previously adsorbed at room temperature during the stabilization period of the experiment under hydrogen flow. In the case of the SO-SRMO treatment, a delay in the H_2_ release is observed, most likely due to the stronger adsorption energy for the Zr-rich pyrochlore monolayer, as shown in Table S1, that could be also responsible for the displacement of the onset of the reduction of cerium from 220°C to 300°C with respect to the MS-TPR experiment (Figure 4). Furthermore, note also that the hydrogen consumption, as measured from the high temperature peak areas, is almost equivalent in both cases to the reduction of the Ce-containing phase. In conclusion, the improvement of the reducibility of the sample at low temperature can be attributed to the presence of the Ce/Zr pyrochlore monolayer in the case of SO-SRMO sample, in good agreement with previously reported results.^18^^,^^20^^,^^21^

### Modeling the surface nanostructures

Surface models were built based on the fluorite structure. The X axis was set along the fluorite-like [2 -1 -1] zone axis, with a length equal to one modulus and the Y axis along the [0 1 -1] direction with length equal to two moduli. In this way the supercell is periodic in both X and Y directions and the area is large enough to avoid interaction between defects or other chemical species from adjacent supercells. The axis is long enough to create (111) surface slab models in vacuum. The slab includes 9 atomic planes, which is equal to 3 fluorite-like layers made of (O-*M*-O) atomic planes, each of them. The bottom layer will be kept fixed, while the other two will be allowed to relax during calculations. The dimensions of the supercells are scaled to the DFT-relaxed lattice parameter for the bulk systems: 5.48 Å and 5.11 Å for CeO_2_ and ZrO_2_, respectively.

The first two models, called zr3 and ce3, consist of a slab made of 3 layers of cubic zirconia (as a first approximation to YSZ) and 3 layers of pure ceria, respectively (see Figures S10A and S10B). The values for the unrelaxed and relaxed zirconia (111) surface energies were estimated to be 1.23 and 0.76 J/m^2^, respectively. The latter value is in good agreement with the literature, but it is slightly lower than the one reported by the authors elsewhere^19^ using ultrasoft pseudopotentials (0.82 J/m^2^). The values for the unrelaxed and relaxed ceria (111) surface energies are calculated in the same way: 0.67 and 0.66 J/m^2^, respectively, in good agreement with previous results.^54^ These data suggest that lattice relaxation plays an important role when Zr is present.

The simulation of the supported ceria (111) monolayer is done considering the same models as for pure ceria or zirconia, but in this case the two bottom layers correspond to zirconia and the top surface layer to ceria, model (zr2ce, see Figure S10C). In order to build this supercell, the ceria lattice parameter was shrunk to match the lattice parameter of zirconia, in good agreement with the experimental data (see Figure 1). Previous DFT calculations, performed with ultrasoft pseudopotentials, were able to demonstrate that this contraction is not a singular feature of the CeO_2_/YSZ system, but, instead, a natural behavior of ceria monolayers.^19^ In the present work, these calculations have been reproduced under the projector augmented wave (PAW) approximation and reached the same conclusion. Using a supercell containing just one unsupported (free standing) monolayer in vacuum, periodic in X and Y directions, and performing a relaxation of the unit cell volume and the atomic positions (keeping fixed the z-coordinates of cerium atoms), the system experiences a planar contraction consistent with a reduction of the ceria lattice parameter to 5.11 Å, which matches that of bulk zirconia. This structural modification is somehow compensated by an increase in the distance between the Ce and O planes inside the layer, with respect to that in bulk ceria. After relaxation of the zr2ce model, the surface ceria monolayer suffers an outward displacement of 0.28 Å. This increment, around 9%, of the last spacing of the (111) planes in the CeO_2_/YSZ is in good agreement with the experimental data (Figure S2C). These modifications of the ceria monolayer with respect to bulk ceria (XY-contraction, Z-expansion …) could partly explain the distinct chemical behavior of supported ceria monolayers.^55^

The pyrochlore (111) monolayer is also epitaxially grown on top of zirconia, as shown experimentally in Figures 2 and 3. Therefore, the simulation of the supported Zr-rich pyrochlore (111) monolayer was done considering a model with the same dimensions than before, but in this case the top surface layer corresponds to a Zr-rich pyrochlore (111) monolayer (model zr2(zr), Figure S10D). For comparison, a model with a Ce-rich pyrochlore monolayer was also built (model zr2(ce), Figure S10E).

The surface energy, calculated under the PAW approximation, for these three models, the supported ceria, the Ce-rich, and the Zr-rich pyrochlore (111) monolayers, were 0.63, 0.48, and 0.58 J/m^2^, respectively. Note that all of them are lower than the (111) surface energy for pure zirconia (0.76 J/m^2^) (see Table S2). These data suggest the formation of a Ce-rich pyrochlore monolayer after an SO treatment. The reason why this phase is not observed experimentally should be found in the difficulty of ordering the cations themselves during the oxidation treatment without the presence of oxygen vacancies. It must be taken into account that oxygen vacancies are responsible for cationic ordering in bulk pyrochlore structure. In any case, these data are in good agreement with the formation of a Ce-rich flourite solid solution, maybe with some degree of cationic ordering, as experimentally found in sample CZ1 after an SO treatment, Figure 1.

To understand the nanostructure resulting from the SRMO treatment (Figures 2 and 3), the same models but considering cerium in its reduced state by introducing oxygen vacancies in the most feasible and stable positions were built, as shown in Figure S11. The surface energy calculated from these three new models, the supported reduced ceria (C-Ce_2_O_3_), the Ce-rich, and the Zr-rich reduced pyrochlore (111) monolayers, are now 1.30, 0.86, and 0.48 J/m^2^, respectively. These data suggest the preference for the formation of a Zr-rich pyrochlore (111) monolayer in sample CZ2 after the SR step. This result is in good agreement with the experimental data and with the following fact: the oxygen vacancies in bulk reduced pyrochlore, Ce_2_Zr_2_O_7_, are accumulated in the Zr-rich (111) layers and none of them in the Ce-rich layers. Therefore, it would be easier to accommodate the oxygen vacancies during the reducing treatment if the created pyrochlore monolayer is the one enriched in Zr. Once the Zr-rich pyrochlore monolayer is created, facilitated by the high temperature treatment and the presence of oxygen vacancies during the reducing step, it is oxidized during the MO step without allowing the cations to move.

### Adsorption and dissociation of the hydrogen molecule

The first aspect to consider in the theoretical study of the interaction of the samples with the reductant during the TPR experiment is the adsorption of molecular hydrogen on the surface. There are two main configurations for this type of interaction in oxides, which have been reported in the literature.^24^ In the first case, the hydrogen molecule locates perpendicular to the surface on top of an oxygen atom. In the second case, hydrogen lays horizontally on top of a cation. In all the calculations performed in this work the most stable configuration corresponds to the second case, with hydrogen lying on top of a cerium atom. Therefore, this was the configuration chosen as the starting point (Figure S12).

In order to take into account the nature of the interaction of molecular hydrogen with the surface, functionals which include van der Waals forces were considered, in particular the vdW-DF2 functional,^56^^,^^57^ which is based on the vdw-DF method.^58^ The values obtained with this functional are two or three times those obtained using normal Perdew-Burke-Ernzerhof (PBE). However the energy trends, as it can be seen in Table S1, do not change very much, as it has been already reported in the literature.^24^ Therefore, the influence of this adsorption energy in the dynamics of the whole process of reduction is negligible.

**Figure 5:**
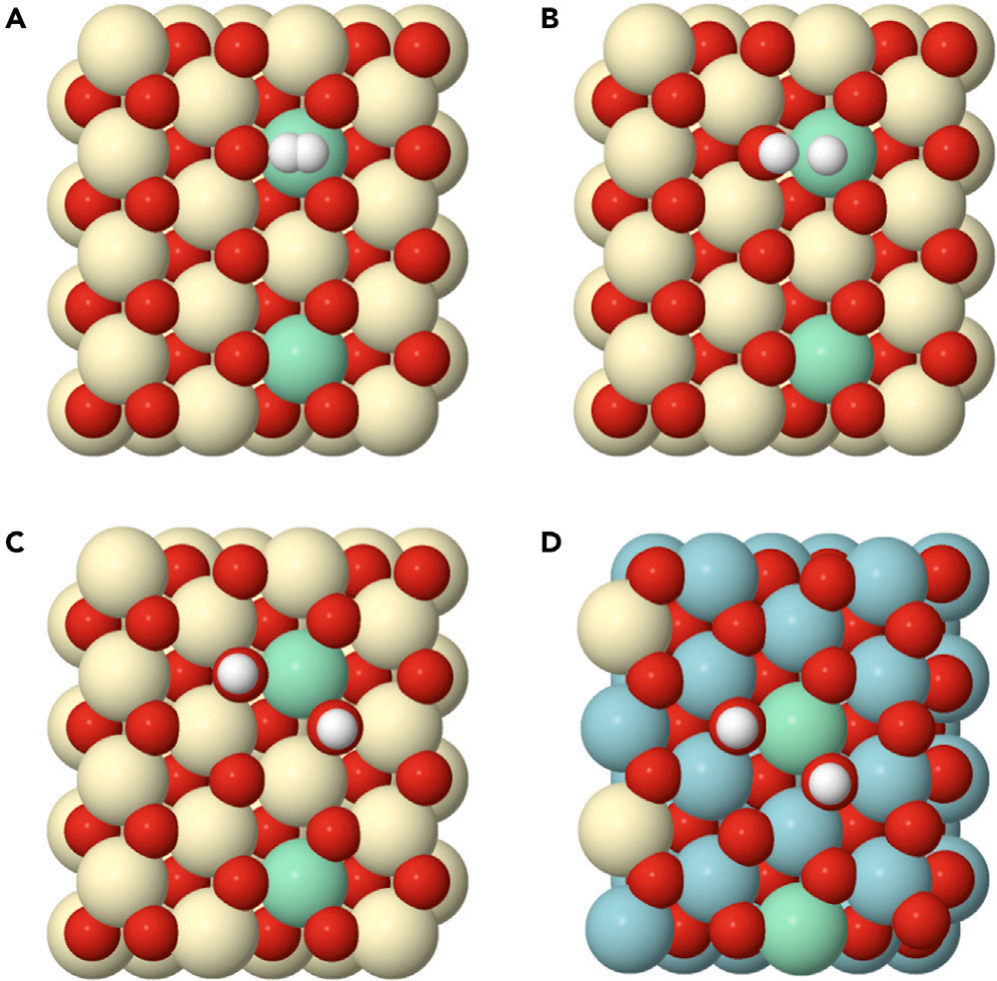
Mechanism for hydrogen dissociation (A) Initial image of the CI-NEB calculation where an adsorbed hydrogen is relaxed on top of a Ce^4+^ cation of a bulk ceria (111) surface, ce3 model. Green cerium atoms are those that are reduced during the formation of the hydroxyl groups. (B) Intermediate image for the ce3 model found to be, after the completion of the CI-NEB calculation, the transition state (TS) for the dissociation reaction. (C) Last image for the ce3 model of the CI-NEB calculation, where two hydroxyl groups are formed and relaxed. The same result is obtained for the zr2ce model. (D) Last image of the CI-NEB calculation for the zr2(zr) model.

The complete process for the dissociation of hydrogen, as shown in Video S1 for the case of a pure ceria (111) surface, can be traced by the climbing image-nudged elastic band (CI-NEB) method, under the spin-polarized DFT+U approximation. The starting point is the adsorbed hydrogen molecule on top of a cerium atom pointing to an oxygen atom (Figure 5A). During the dynamical calculation, the H_2_ molecule moves toward the oxygen atom, weakening the bonding between the two hydrogens. The transition state, determined by the CI-NEB method, is an intermediate stage of the path with the highest energy, where a hydroxyl group and a cerium hydride are almost formed, but there is still some interaction between the two hydrogen atoms (see Figure 5B). The process follows with the breakage of this transition state. One hydrogen is retained as a hydroxyl group, and the second one approaches another oxygen nearby. During this process the reduction of two cerium atoms is taking place. Therefore, the final situation corresponds to two hydroxyl groups and two reduced cerium atoms (Figure 5C). The Ce^3+^ configuration chosen for the calculations^23^ allows for a comparison between the different models. These results are in good agreement with the literature,^22^^,^^24^^,^^28^ which support a homolytic dissociation of the hydrogen molecule, in the particular case of reducible oxides, like CeO_2_. The local minima corresponding to the heterolytic dissociation, as reported elsewhere,^25^^,^^26^^,^^27^ have not been followed, for simplicity, in these calculations.

Video S1. Hydrogen dissociation, for the pure ceria (111) surface, model ce3, related to Figure 5 and Table 1

The mechanism of dissociation for the models zr2ce and zr2(zr) is almost identical to the one described for the pure ceria (111) surface. Thus, Figure 5D shows the final step for the last one. The energy required for hydrogen dissociation can be calculated from the energies of the first and last steps. These values are gathered in Table 1 for the different models. The lowest dissociation energy is observed for the pure ceria (111) surface, model ce3, whereas the highest one corresponds to the supported (111) ceria monolayer, model zr2ce.

**Table 1:** Energies (in eV) of the different elementary steps involved in the hydrogen molecule dissociation for the different models

E (eV)	ce3	zr2ce	zr2(zr)
E_f_(2OH^−^)	−2.70	−1.20	−2.44
E_A_(TS)	0.67	1.58	0.78
E_A_(H^+^-H^-^)	0.80	1.51	0.76

Ef(2OH^−^) corresponds to the enthalpy of this process, where two hydroxyls groups are formed. EA(TS) is the activation barrier for this process, calculated by the CI-NEB method. EA(H+-H-) corresponds to the energy calculated using the frozen heterolytic model.

The trend observed for the activation energy of the hydrogen dissociation reaction provides also key information (Table 1). Note first that the supported ceria (111) monolayer appears as the least reactive structure, with an associated activation energy more than twice that of pure ceria. On the other hand, the activation barrier for the Zr-rich supported pyrochlore (111) monolayer structure is slightly higher than that of the pure (111) ceria surface. So, hydrogen activation does not provide an explanation to the improved reducibility in hydrogen of the supported pyrochlore nanostructure with respect to that of pure ceria. To clarify this question, a more complete analysis of the whole reduction process is necessary.

In this respect, it is important to highlight that X-ray photoelectron spectroscopy (XPS) studies of high surface area ceria samples^59^ have reported that reduction in hydrogen at 260°C (533 K) generates 28% of Ce^3+^ species without the formation of a significant amount of oxygen vacancies, leading to an O/Ce ratio very close to 2. Likewise, when the reduction temperature rises up to 400°C (673 K), the percentage of Ce^3+^ increases to 34%, but once more just a small decrease of the O/Ce ratio is detected. Importantly, the first H_2_O evolution signal in the corresponding TPR experiment peaks at 434°C (707 K). Therefore, these data^59^ indicate that the hydroxylation of the surface, and therefore, ceria surface reduction, occurs long before the formation of oxygen vacancies, probably at temperatures below 260°C (533 K). This, in turn, suggests that the reducibility differences between pure ceria and the YSZ-supported pyrochlore (111) monolayer, considered as the formation of oxygen vacancies, are limited not by the activation barrier for hydrogen dissociation but, instead, by a different process, as shown in the next section.

As a first approximation, the transition state for heterolytic hydrogen dissociation can be modeled by just positioning a hydrogen atom on top of one oxygen and the second one on top of the neighboring cerium atom and allowing them to move just in the direction perpendicular to the surface (Z direction). The energy calculated using the PBE functional of this model is very close to the calculated transition state using the NEB method and the PBE+U functional (Table 1, last row). It is important to understand that this model, which corresponds to a hypothetical “frozen” heterolytic breakdown, does not include any cerium atom in its reduced state. The hydrogen molecule is split in a proton H^+^ bound to the oxygen to form a hydroxyl group and a hydride H^−^ interacting with the cerium atom (Ce^4+^) beneath. Data obtained through this model can be used as a reference parameter to estimate the energy barrier of H_2_ dissociation in a fast way.

In this context, it is also interesting to calculate the Bader charges of the main atoms involved in the reaction. For example, in the heterolytic approximation model for the pure ceria (111) surface, the Bader charges for both hydrogens amount to 0.40 and 1.40, indicating that one is acting as H^+^ and the second one as hydride. The Bader charge of the cerium atom just beneath the second hydrogen is 9.77, which indicates some accumulation of charge on this cation (a typical value for Ce^4+^ in these oxides is 9.64). The corresponding values for the transition-state model are very similar: 0.34, 1.33, and 9.79, which demonstrates the similarity between the two models. However, the Bader charges calculated for the two hydrogens in the following steps of the CI-NEB path, after the transition state, amount to 0.34 and 0.35, which indicates the formation of two hydroxyl groups. In this case the Bader charges for the two Ce^3+^ and the rest of Ce^4+^ present in the model are 9.87 and 9.64, in good agreement with the values reported elsewhere for these two types of cations.^60^ From these data, it becomes clear that the ability of cerium atoms to be reduced drives the hydrogen dissociation through the homolytic route.

### Formation and desorption of the water molecule

**Figure 6:**
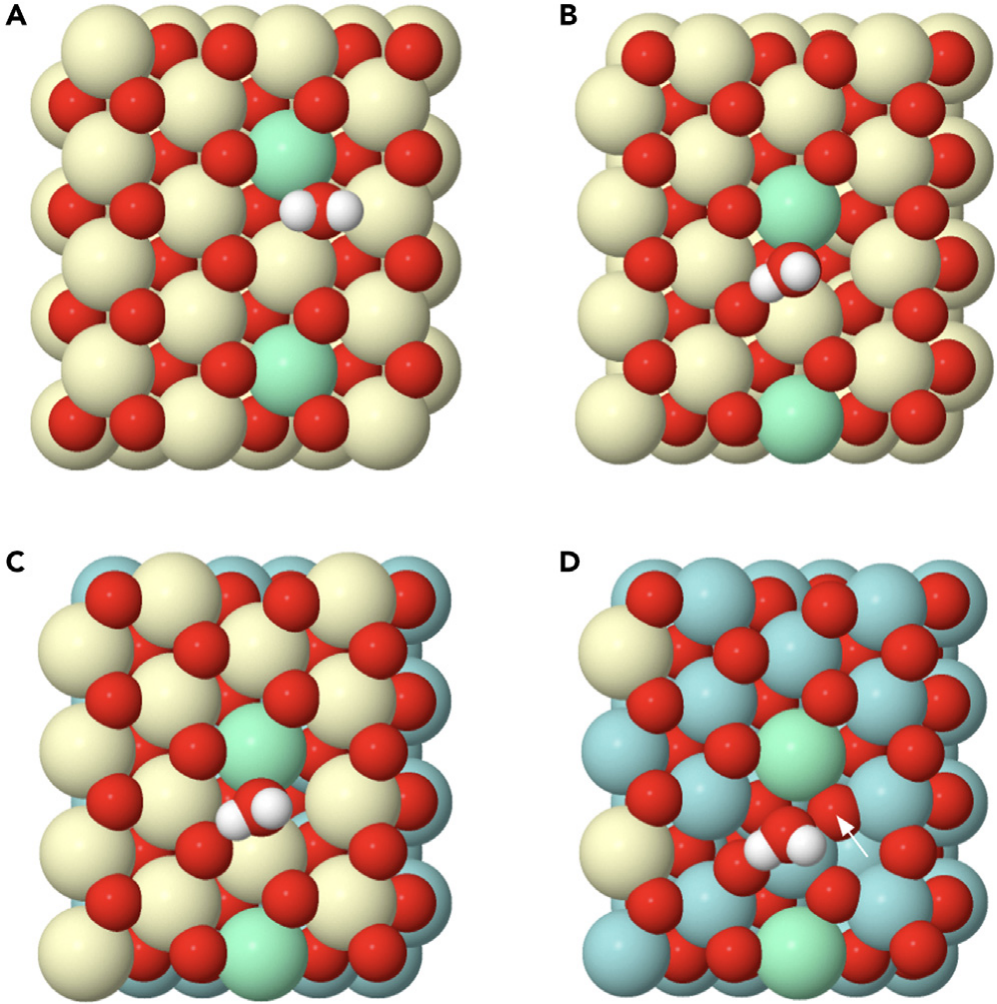
Models containing an adsorbed water molecule Green atoms correspond to Ce^3+^. (A) Bulk ceria (111) surface, ce3 model, before relaxation. (B) Bulk ceria (111) surface, ce3 model, after relaxation. (C) Supported ceria (111) monolayer, zr2ce model, after relaxation. (D) Supported Zr-rich terminated pyrochlore (111) monolayer, zr2(zr) model, after relaxation. The white arrow points at the oxygen that has moved up from the subsurface.

The following step, after hydroxylation, is the formation of water. These water molecules will remain adsorbed on the surface of the oxide and later on released. As shown here, the formation of adsorbed water is a critical process. One hydrogen should move, breaking one hydroxyl bond, and connect to a neighboring hydroxyl. Therefore, the last step for the CI-NEB calculation requires finding first the relaxed structure for the adsorbed water molecule. For that, a supercell was built for the pure ceria model approaching two hydrogens to one surface oxygen (0.96 Å) forming an angle close to the one observed in the water molecule (Figure 6A). After relaxation, the water molecule moves up and leaves the oxygen position but still remains adsorbed on the surface (Figure 6B). The oxygen of the water molecule bridges two cerium atoms (one Ce^4+^ and one Ce^3+^), and one of the two hydrogens moves to an adjacent oxygen atom (see Video S2). One of the O-H distances in the water molecule is 0.97 Å, which is expected for the O-H bond in an isolated water molecule. However, the second one is a little bit longer (1.04 Å), very likely because that hydrogen interacts by a weak hydrogen bonding with an adjacent oxygen. The distance of this hydrogen bond is 1.64 Å, which is a value larger than that found in typical hydrogen bonds (1.25 Å). The complete path from the two hydroxyl groups to the adsorbed water molecule can be viewed in Video S3. The result found for the supported ceria monolayer, zr2ce model, is very similar (Figure 6C).

Video S2. Adsorbed water relaxation, for the pure ceria (111) surface, model ce3, related to Figure 6 Last step considered for the CI-NEB calculation

Video S3. Adsorbed water formation from two hydroxyls, for pure ceria (111) surface, model ce3, related to Figure 6 and Table 2

Besides this, the major difference was found in the zr2(zr) model. In this case, at the same time that the water molecule is being formed and moving up, the associated oxygen vacancy is occupied by a subsurface oxygen atom, as marked with a white arrow in Figure 6D. The movement of this oxygen pushes the water molecule up and creates a subsurface vacancy which is coordinated to four zirconium atoms (Video S4). This is the natural vacancy found in bulk reduced Ce_2_Zr_2_O_7_ pyrochlore. At the end of this process, the water molecule rotates forming a hydrogen bond with a near-surface oxygen atom.

Video S4. Adsorbed water formation from two hydroxyls, for the supported Zr-rich pyrochlore (111) monolayer, model zr2(zr), related to Figure 6 and Table 2 Note the subsurface oxygen movement.

The structural relaxation induced by the subsurface oxygen movement, in the zr2(zr) model, leads to a value almost zero (0.02 eV) for the energy of formation of the water molecule from the two hydroxyl groups. On the other hand, the activation energy of this process is also very low (0.22 eV), which means that the formation of adsorbed water from the hydroxyl groups does not have a thermodynamic or kinetic constraint in clear contrast with the other models for which this value is equal or above 1.38 eV (Table 2). The specific structure of the pyrochlore monolayer allows the movement of the subsurface oxygen which, in turn, boosts the formation of water. The movement of this oxygen is the feature that makes the difference, and it is in fact the key to explain the enhancement of the reduction behavior of the Zr-rich pyrochlore surface. Underlying this movement is the tendency for the oxygen vacancy to settle in a 4-fold Zr coordinated position, reducing the Zr coordination number.

**Table 2:** Energies (in eV) involved in the formation and release of the water molecule. E_f_(H_2_O) corresponds to the formation energy of an adsorbed water molecule from two adjacent hydroxyls groups

E (eV)	ce3	zr2ce	zr2(zr)
E_f_ (H_2_O)	1.30	1.45	0.02
E_A_ (H_2_O)	1.38	1.55	0.22
E_d_(H_2_O)	0.75	0.92	0.53

E_A_(H_2_O) is the energy barrier of this process, calculated using the CI-NEB method. E_d_(H_2_O) corresponds to the water desorption energy.

**Figure 7:**
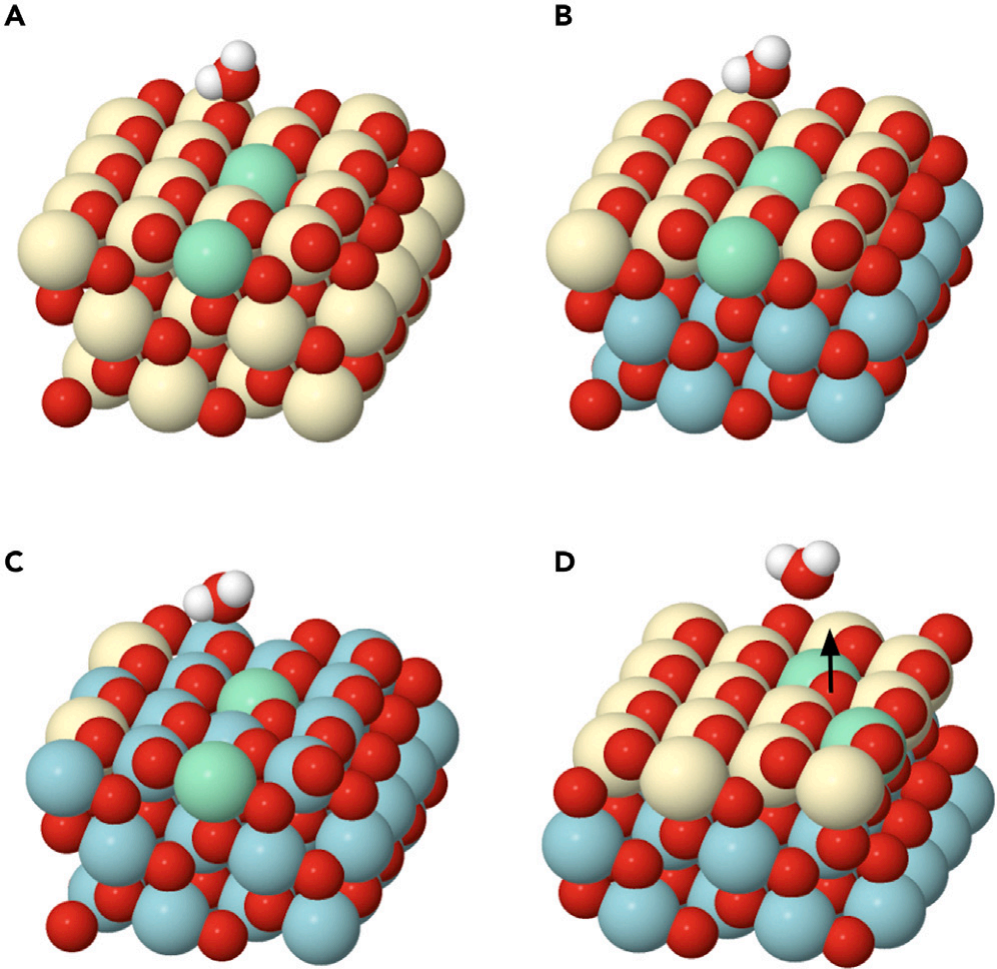
Relaxed models containing the desorbed water molecule, 4–5 Å away from the surface (A) For a bulk ceria (111) surface, ce3 model. (B) For a supported ceria (111) monolayer, zr2ce model. (C) For a supported Zr-rich pyrochlore (111) monolayer, zr2(zr) model. (D) For a supported ceria (111) monolayer, from zr2ce model, but the Ce^3+^ are in a different configuration. The black arrow points at the oxygen that has moved up from the subsurface.

Once the water molecule is formed, its desorption constitutes the final step of the reduction process, e.g., Videos S5 and S6. This step was modeled through the CI-NEB method, by moving the molecule up in the Z direction (Figure 7). In general, this step requires an input of energy and there is not a transition state. In the case of pure ceria the value (+0.75 eV) is close to that reported for water physisorption near to a vacancy site^61^ (Table 2). Again, this value is higher for the case of the supported ceria monolayer, model zr2ce, and is lower for the Zr-rich pyrochlore monolayer, model zr2(zr).

Video S5. Water desorption, for the pure ceria (111) surface, model ce3, related to Figure 7 and Table 2

Video S6. Water desorption, for the supported Zr-rich pyrochlore (111) monolayer, model zr2(zr), related to Figure 7 and Table 2

**Figure 8:**
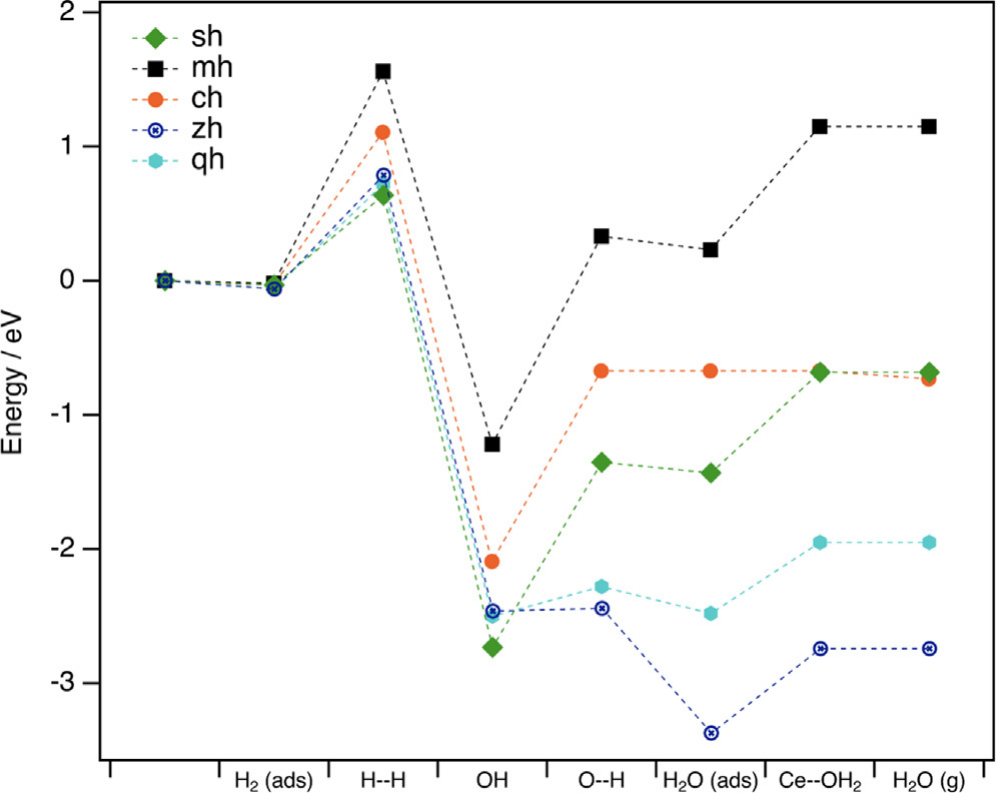
Graphical representation of the energies involved in each step of the reduction reaction, starting from the non-adsorbed hydrogen molecule and ending in the desorbed water molecule The following models are considered: (sh) bulk ceria (111) surface. (mh) supported ceria (111) monolayer. (ch) supported Ce-rich terminated pyrochlore (111) bilayer. (zh) supported Zr-rich terminated pyrochlore (111) bilayer. (qh) supported Zr-rich pyrochlore (111) monolayer.

Figure 8 shows a graphical representation of all the steps for the different models. Note that there are three key steps in the formation of water from hydrogen. The first one corresponds to hydrogen dissociation, the second one, the adsorbed water formation, and the last one, the desorption of water. In each model, the activation energy associated with at least one of the first two steps is higher than that of water desorption, so the third and last step do not seem to be critical, and the differences should be found in the first two steps.

In the pure ceria (111) surface, the second step is clearly the limiting factor, with an activation energy of 1.38 eV (being the activation energy of the first step 0.67 eV). In the supported ceria monolayer, both steps are limiting; the first one is higher (1.58 eV), but the second one is very close (1.55 eV). The most significant result appears in the supported Zr-rich pyrochlore monolayer, model zr2(zr), in which the first step is clearly the limiting factor (0.78 eV). In fact, this is the only model where the second step (activation energy 0.22 eV) clearly does not play any control on the reaction. This is due to the movement of the subsurface oxygen while the adsorbed water is forming. The comparison of the activation barriers values of the limiting steps of the ce3 model (1.38 eV), the zr2ce model (1.58 eV), and the zr2(zr) model (0.78 eV) indicates that the movement of the subsurface oxygen is the main factor responsible for the enhancement of the low-temperature reducibility of the CZ2 sample after an SRMO treatment.

The activation barriers calculated in this work are relevant to understand the differences in the values of the onset temperature for the reduction in hydrogen of the systems after a thermal treatment. Once the reduction process starts, other parameters should be taken into account, like the effects of the oxygen vacancies, hydrogen coverage degree, or the formation of surface or bulk hydride species.^59^^,^^62^^,^^63^^,^^64^^,^^65^

### Extending the results to other ceria loadings and bulk mixed oxides

The results obtained for the Zr-rich pyrochlore (111) monolayer can be easily applied to other systems. For instance, some calculations have been performed with a Ce/Zr monolayer with a lower content of cerium (lower than 25/75) and the energies involved are very similar to the perfect Zr-rich pyrochlore (111) monolayer, again due to the movement of the subsurface oxygen during the adsorbed water formation (Video S7). The presence of isolated cerium atoms embedded in the surface is crucial. The formation of a cluster of just 3 cerium atoms prevents the movement of subsurface oxygen (Video S8). In the Zr-rich pyrochlore (111) monolayer, all the cerium atoms are surrounded by just one zirconium atom. Therefore, this nanostructure represents the highest concentration of embedded atomically dispersed cerium atoms in a zirconia (111) surface. In this way, the formation of the Zr-rich pyrochlore monolayer is an easy and direct way to create cerium SACs.

Video S7. Adsorbed water relaxation, for zirconia (111) surface model with two isolated cerium atoms, related to Figure 6 Note the subsurface oxygen movement.

Video S8. Adsorbed water relaxation, for zirconia (111) surface model with a three cerium atoms cluster, related to Figure 6

When the content of Ce loading is increased to 0.7 monolayers, the amount of Ce in the surface monolayer after an SRMO treatment does not increase significantly, as seen experimentally in this work. When the Ce loading is higher than that, some nuclei of pyrochlore phase can be created in the form of a nanoparticle or an ultra-thin layer.^18^^,^^21^ As a simple model for the pyrochlore nucleus, a zirconia-supported pyrochlore (111) bilayer was built. Two possibilities are found for this system due to the alternation of Ce- and Zr-rich (111) planes of the structure of the pyrochlore: a bilayer ended in a Ce-rich (111) surface, model zr(zrce), or ended in a Zr-rich (111) surface, model zr(cezr), as can be seen in Figure S13. The complete set of results corresponding to these new models, collected in Table S3, have been added to Figure 8. The activation energy values found for hydrogen dissociation and water formation on the Ce-rich model are close to those of the supported ceria monolayer. However, for the Zr-rich case, these values are closer to those of the Zr-rich pyrochlore monolayer. This suggests that the reducibility of the system at low temperature is enhanced when the pyrochlore phase ends in a Zr-rich (111) surface, regardless of the thickness of the pyrochlore nucleus.

The calculated surface energy for both zirconia-supported pyrochlore bilayers are 0.67 and 1.06 J/m^2^, for the Ce-ended and Zr-ended cases, respectively. These data may suggest the preference of the system to expose a Ce-terminated surface. But, taking into account that the pyrochlore phase forms during the reduction step of the SRMO treatment, when all the Ce atoms should be reduced, two new models zr(zrce)∗ and zr(cezr)∗, based on the reduced Ce_2_Zr_2_O_7_ phase, must be considered (Figure S14). Values of 1.21 J/m^2^ and 0.62 J/m^2^ were obtained in this case for the Ce-rich and Zr-rich surface terminations, respectively (Table S4). Interestingly, under reducing conditions the Zr-rich termination becomes the most stable, in good agreement with the results obtained for the pyrochlore monolayer case. These calculations explain the preference for a Zr-rich surface termination of the pyrochlore nuclei after an SRMO treatment, as evidenced by the experimental HR-STEM studies.^44^ The pyrochlore nucleus is created during the SR step ending in Zr-rich surfaces, and, later on, the system is oxidized at low temperature (MO step) not allowing the cations to move. These results can be extended to pyrochlore nuclei of different sizes and morphologies exposing (111) facets, like the one found in Ce/Zr bulk-type mixed oxides after an SRMO treatment,^43^^,^^47^ e.g., Figure S15.

Finally, it is worth mentioning that the movement of the subsurface oxygen was also found during the calculation of the reduction in hydrogen in the case of the supported Ce-rich pyrochlore bilayer and the ceria monolayer, but this happens during the last step (water desorption) in both cases, not affecting the total reduction process (Figure 7D and Video S9). On the other hand, this oxygen movement was also found to happen spontaneously during the relaxation of certain surface vacancies. This is the case for the supported ceria monolayer in a specific configuration for the position of the Ce^3+^ ions (Video S10), the Ce-ended pyrochlore bilayer, where a double movement of oxygens takes place (Video S11), and the Zr-ended pyrochlore bilayer/monolayer (Video S12). In all these cases, the extra relaxation energy for the vacancy formation due to the movement of the oxygen amounts to 1 eV approximately. Besides this, the minimum vacancy formation energy is found for the Zr-rich pyrochlore monolayer (0.1 eV) (see Table S5), making this nanostructure again the most reducible, this time from the thermodynamic point of view.

Video S9. Water desorption, for the supported ceria (111) monolayer, model zr2ce, related to Figure 7 Two Ce3+ cations are in a different configuration. Note the subsurface oxygen movement.

Video S10. Relaxation of a surface vacancy, for the supported ceria (111) monolayer, model zr2ce, related to Figure 6 Two Ce3+ cations are in a special configuration. Note the subsurface oxygen movement.

Video S11. Relaxation of a surface oxygen vacancy, for the supported pyrochlore (111) bilayer with Ce-rich surface, model zr(zrce), related to Figure 6 Note the movements of two subsurface oxygens.

Video S12. Relaxation of a surface oxygen vacancy, for the supported Zr-rich pyrochlore (111) monolayer, model zr2(zr), related to Figure 6 Note the movement of the subsurface oxygen.

### Conclusions

The structure and the interaction with hydrogen of the CeO_2_/YSZ system (ceria loading equal or less than one monolayer) submitted to different redox pre-treatments have been investigated.

After an SO treatment, the ceria phase tends to wet the YSZ surface in the form of one monolayer of a Ce-rich Ce/Zr solid solution oxide. The calculated activation energy for hydrogen dissociation and water formation on this nanostructure is very high, which explains the worsening of the reducibility of the system after this treatment, with respect to pure ceria (111) surface.

After an SRMO treatment, the reaction between ceria and the YSZ support leads to a pyrochlore phase that also spreads onto the surface of the YSZ as a Zr-rich monolayer, regardless of the initial ceria loading (less than one monolayer), while ceria excess accumulates as 3D particles. The limiting step for reduction in this case is hydrogen dissociation, whose activation energy is lower than that observed in bulk ceria (111) surfaces. This provides fundamental support to the reducibility enhancement of this system in hydrogen after an SRMO treatment, with respect to pure ceria.

From a mechanistic perspective, the particular interface formed between zirconia and the supported pyrochlore phase allows the movement of oxygen from the subsurface to the surface vacancy just formed during the hydroxyl condensation step. In the case of the Zr-rich pyrochlore surface, this movement significantly decreases the activation energy and boosts water formation. This is a key to fully rationalize the reducibility of these systems after an SRMO treatment.

The calculation of the whole set of activation energies evidences that a Zr-rich pyrochlore (111) monolayer is even more convenient than a Zr-ended pyrochlore (111) bilayer, or any other larger Zr-ended pyrochlore nucleus, for reduction by hydrogen at low temperature. It is possible to prepare a system containing just one Zr-rich pyrochlore monolayer by depositing an amount of ceria equivalent to ¼ monolayer on top of YSZ. A close inspection of the Zr-rich pyrochlore (111) monolayer structure reveals that this situation corresponds to a close-packed disposition of isolated Ce^4+^ cations within one (111) zirconia surface. Therefore, the formation of the Zr-rich pyrochlore surface represents the most convenient way to create cerium SACs with improved redox performance.

Importantly, it has been quantitatively demonstrated that it is possible to decrease the amount of lanthanide content to reach a state of a surface single cation (SC) dispersion without losing, but rather improving, redox properties. Moreover, a subtle movement of subsurface oxygen during the reduction process is the key factor to understand such an improvement.

### Limitations of study

The reducibility of the samples was studied using a flow of H_2_(5%)/Ar during programmed thermal reduction (TPR) experiments. In order to understand the redox response of catalysts under real working conditions, operando experiments must be carried out for each specific reaction.

## STAR★Methods

### Key resources table

**Table:** 

REAGENT or RESOURCE	SOURCE	IDENTIFIER
**Software and algorithms**		

Rhodius	University of Cadiz	http://temserver.uca.es
Eje-Z	University of Cadiz	http://temserver.uca.es
Quantum ESPRESSO	Quantum ESPRESSO Foundation	https://www.quantum-espresso.org/
TEM-SIM	Earl J. Kirkland	https://sourceforge.net/projects/computem/
Mu-STEM	L. J. Allen et al.	https://github.com/HamishGBrown/MuSTEM

**Chemicals, peptides, and recombinant proteins**		

YSZ	Sigma-Aldrich	CAS 114168-16-0
Ce(NO_3_)_3_·6H_2_O	Sigma-Aldrich	CAS 10294-41-4
NH_3_ (32%)	VWR	CAS 1336-21-6
O_2_(5%)/He	Air Liquide	Ref. P3350L50S2A001
H_2_(5%)/Ar	Air Liquide	Ref. DR302059
He	Linde	Ref. 3340152

**Other**		

Double-corrected Scanning Transmission Electron Microscope	Thermo Fisher Scientific	FEI Titan Cubed Themis 60-300 microscope
Scanning Transmission Electron microscope	Thermo Fisher Scientific	FEI Talos F200X G2 microscope
Microwave oven	Milestone	Ethos One
Electron Energy Loss Spectroscopy (EELS) detector	Gatan	HR Quantum Gatan ERS Energy Filter
Mass Spectrometer	Pfeiffer Vacuum	Prisma
Temperature Programmed Reduction (TPR)	Micromeritics Instrument Corp.	AutoChem II
Lacey/Carbon grids	Aname S.L.	LC325-CU25

### Resource availability

#### Lead contact

Further information and requests for resources and reagents should be directed to and will be fulfilled by the lead contact, José A. Pérez-Omil (jose.perez-omil@uca.es).

#### Materials availability

This study did not generate new unique reagents.

### Method details

#### Experimental

The samples were prepared using yttria-stabilized zirconia (YSZ, 8 at% Y) from Sigma-Aldrich (CAS number 114168-16-0). This support was treated in air in a muffle oven at 900°C for 2 h in order to increase its textural stability. After that, the percentage of {111} faces present in this support is approx. 75%. The final specific surface area was 33 m^2^/g.

A sample, labeled as CZ1, was prepared by incipient wetness impregnation (IWI)^66^ using 1 g of the YSZ support and an aqueous solution 0.2 M of Ce(NO_3_)_3_·6H_2_O. After three impregnation cycles (pore volume equals to 0.47 mL/g), the sample is dried in an oven at 80°C and further submitted to a treatment in air at 400°C for 1 h. The deposited amount of CeO_2_ (50.23 mg) was the one required to cover the surface of the support with roughly 0.71 monolayers, which corresponds to a concentration of 3.71 wt % of cerium atoms in good agreement with ICP measurements. On the other hand, this concentration can be expressed in terms of cationic (Ce, Zr, Y) fraction, 3.26 at%, thus showing the low content of lanthanide elements present in the sample.

A second sample, CZ2, was prepared by the microwave hydrothermal method in order to get a better dispersion of the ceria phase on top of the YSZ sample. An amount of 63.8 mg of Ce(NO_3_)_3_·6H_2_O is dissolved in 50 mL of distilled water. Next, 1 g of the calcined YSZ support is added and the suspension is stirred for 20 min. The pH is regulated to 10 using drops of NH_3_ (32%). The mixture is transferred to a 100 mL Teflon reactor of the Ethos One microwave oven. After a hydrothermal treatment at 180°C for 30 min the sample was cooled, filtered, dried and treated in a muffle furnace for 2 h at 500°C. A loss of ceria (36%) during the hydrothermal treatment was detected from an statistical analysis of XEDS experiments at low magnification using the FEI Talos F200× G2 microscope. The actual Ce loading in this sample, 1.14 at% of Ce atoms, corresponds to 0.24 monolayers of ceria on top of YSZ.

Both samples were submitted to several thermal treatments, using in all cases a temperature ramp of 10°C/min and a 60 cm^3^/min flow. The so-called Strong Oxidation (SO) treatment consisted in heating a portion of the sample up to 900°C under O_2_(5%)/He flow, keeping the temperature for 2 h and cooling the sample under the same flow. The Strong Reduction Mild Oxidation (SRMO) treatment consisted of heating a portion of the sample up to 900°C under H_2_(5%)/Ar flow, keeping the temperature for 2 h, cooling it down to 500°C changing the flow to pure He, keeping the temperature for 1 h to remove the adsorbed hydrogen and cooling it down to room temperature, completing in this way the SR step. After this, the sample was cooled further down, using an acetone trap, and the flow changed now to O_2_(5%)/Ar to allow for a smooth reoxidation. The sample was heated again up to 500°C in O_2_(5%)/Ar flow, kept at this temperature for 1 h and cooled down to room temperature under the same flow, completing the MO step. Combinations are possible, like the SO-SRMO treatment.

The samples were deposited on lacey carbon supported copper grids for characterization using an aberration corrected FEI Titan Cubed Themis 60-300 operating at 200 keV. The sub-angstrom resolution High Angle Annular Dark Field (HAADF) images were recorded in Scanning Transmission Electron Microscopy (STEM) mode (spherical aberration C_s_, 0.001 mm; fifth-order spherical aberration, 5 mm; defocus, 1 nm; convergence semi-angle, 19.4 mrad). Electron Energy Loss Spectroscopy (EELS) elemental maps were built by collecting the Ce-M_4,5_ signal on a High Resolution Quantum Gatan ERS Energy Filter using the following experimental parameters: energy dispersion, 0.25 eV/channel; collection semiangle, 79.0 mrad; convergence semiangle, 33.7 mrad; exposure time, 100 ms; and probe current, 60 pA. Energy-dispersive X-ray spectroscopy (XEDS) elemental maps were recorded using the Super X-G2 detectors of the microscope. A probe current of 150 pA and a dwell time per pixel around 200 ***μ***s were used. The Ce-L and Zr-K signals were used to map the distribution of the two metals in the samples. Complementary studies were performed at low magnification using a FEI Talos F200× G2 microscope operating at 200 keV, with C_s_ 1.5 mm in STEM mode.

#### Theoretical calculations

The DFT calculations included in this work have been performed using the plane-wave code Quantum Espresso^67^ with the spin polarized Perdew-Burke-Ernzerhof functional (PBE) and taking into account the Projected Augmented Wave (PAW) method. In the case of Zr, O and H the appropriate PAW scalar relativistic pseudopotentials were used.^68^ For Cerium the PAW pseudopotential (called from now on as pspCe4) available in the Rare Earth database from Columbia University is chosen, where 12 electrons are not considered in the core, including the 4f electron.^69^ Self-consistent calculations of the Hubbard U parameter for Ce-4f states in the CeO_2_ structure for this pseudopotential were performed following the implementation of the density-functional perturbation theory available in the HP code,^70^^,^^71^ which provided, after convergence, a value of 3.7 eV. After a complete set of calculations varying the value of the Hubbard U parameter, in order to better adjust the electronic structure of trigonal-Ce_2_O_3_ and the energy of reduction of CeO_2_ the value for U finally considered in this work was 3.5 eV. The convergent wave function cut-off was calculated to be 50 Rydberg. For K-point integrations a 8 × 8 × 8 Monkhorst–Pack grid was used for calculations of the unit cells of bulk systems, while 4 × 4 × 2 grid, 2 × 2 × 1 grid or just gamma point was used for the calculations using supercells, depending on size. For the relaxation processes the Broyden–Fletcher–Goldfarb–Shanno (BFGS) algorithm for geometry optimization included in Quantum Espresso code was used.

One of the main challenges in theoretical calculations applied to oxides is to find a good estimation of the chemical potential of the oxygen molecule. For this reason in the present work this value is calculated using a semi-empirical approximation based on an auxiliary system like Mg/MgO.^72^ Taking into account the Standard Enthalpy of Formation of MgO (−601.6 kJ/mol) it is possible to calculate a value for the energy associated to the oxygen atoms that can be used in other calculations involving oxygen at the same conditions.

E(O)=E(MgO)−E(Mg)−Enthalpy(MgO)

Using this methodology in the case of reduction of the oxidized pyrochlore Ce_2_Zr_2_O_8_ phase to pure pyrochlore Ce_2_Zr_2_O_7_ a value of 108 kJ/mol (1.12 eV per O atom) is obtained, following the equation:

$$\Delta H_{red}=E\left({Ce}_{2}{Zr}_{2}O_{7}\right)+E\left(O\right)-E\left({Ce}_{2}{Zr}_{2}O_{8}\right)$$

This value is higher than the theoretical value previously reported for kappa phase (0.72 eV) but is lower than the experimental value based on electrochemical measurement (1.91 eV calculated from the free energy of reduction at 373 K).^73^

An alternative PAW pseudopotential (called from now on as pspCe3) has also been used where the 4f electron is included in the core (just 11 valence/semicore electrons).^68^ This pseudopotential has been used to simulate Ce^3+^ species while relaxing the structure using just the PBE functional, as a previous step to the final relaxation (performed with the spin polarized PBE+U approximation and the pspCe4 pseudopotential). In order to test the quality of the pspCe3 pseudopotential, the Energy of Formation of Ce_2_Zr_2_O_7_ from the pure oxides (Ce_2_O_3_ & ZrO_2_) has been calculated using this pseudopotential. The obtained value, −118.6 kJ/mol, is very close to the reported experimental measurement,^74^, -121.1 kJ/mol.

The Surface Energy attributed to the built nanostructures used in this work have been calculated following the equation:

$$S_{nano}=1/A\left(E_{supercell}-E_{bulk}-E_{bottom}\right)$$

where A corresponds to the surface area, E_supercell_ to the calculated energy of the relax model, E_bulk_ to a summation of the corresponding units of bulk binary oxides energies (ZrO_2_, CeO_2_ or C-Ce_2_O_3_), and the E_bottom_ to the energy of the bottom zirconia unrelaxed surface.

#### Modeling and STEM simulations

All the supercells for STEM simulations and DFT calculations were built using Rhodius software,^75^ which allows to control the orientation, morphology, and atomic positions of the nanostructures, available on-line on the TEMserver.^76^ Animations were represented using JSmol.^77^ Atomic resolution images were interpreted using Eje-Z software.^78^

Cs-corrected HAADF STEM images were performed with TEMSIM and ***μ***STEM codes.^51^^,^^79^ XEDS/EELS elemental map simulations were performed using ***μ***STEM code considering the Quantum Excitation of Phonons (QEP) approximation.^51^ The following parameters were considered for the calculations: high voltage, 200 kV; third-order spherical aberration, 0.001 mm; fifth-order spherical aberration, 5 mm; Scherzer defocus, 1.9 nm; and HAADF Detector Geometry, 80–200 mrad. A convergence semi-angle of 16 mrad was considered for XEDS elemental map simulations, and 33.7 mrad for the EELS ones. A collection semi-angle of 79.0 mrad was taken into account to calculate the EELS signals. A Gaussian blur with a sigma value of 2 and electronic noise with a standard deviation of 25% were applied to the simulated images. XEDS Ce-L and Zr-K and EELS Ce-M_4,5_ signals were calculated in the simulations.

## Data Availability

• Data reported in this paper will be shared by the lead contact upon request. • This paper does not report original code. • Any additional information required to reanalyze the data reported in this paper is available from the lead contact upon request.
