# Supplementary material for: Supported Ce/Zr pyrochlore monolayers as a route to single cerium atom catalysts with low temperature reducibility
Source: iScience. 2023 Jul 28;26(9):107506. doi: 10.1016/j.isci.2023.107506 (PMC10448079; doi:10.1016/j.isci.2023.107506)
Supplement: Document S1. Figures S1–S5 and Tables S1–S5 [file mmc1.pdf]

iScience, Volume *26*

Supplemental information

Supported Ce/Zr pyrochlore monolayers as a route to single cerium atom catalysts with low temperature reducibility

Jose M. Montes-Monroy, Ramón Manzorro, Lidia E. Chinchilla, William E. Celín, Jose J. Calvino, and Jose A. Pérez-Omil

SUPPLEMENTAL INFORMATION

Departamento de Ciencia de los Materiales e Ingeniería Metalúrgica y Química Inorgánica, Facultad de Ciencias, Universidad de Cádiz, 11510-Puerto Real, Spain

Correspondence: jose.perez-omil@uca.es

1.- Tables

eV / H atom	ce3	zr2ce	zr(zrce)	zr(cezr)
E_{PBE} (eV/H_2)	-0.016	-0.009	-0.016	-0.031
E_{DF2} (eV/H_2)	-0.044	-0.038	-0.046	-0.058
$d_{O\text{-}H}$ (Å)	2.87	3.04	2.94	2.82
$d_{Ce\text{-}H2}$ (Å)	3.10-3.15	3.55-3.62	3.26-3.30	2.93-2.96
$d_{H\text{-}H}$ (Å)	0.75	0.75	0.75	0.75

Table S1.- Data corresponding to the adsorption of the hydrogen molecule, related to Figure 5. Considered cases: the ceria free (111) surface, ce3; the supported ceria monolayer, zr2ce; the supported Ce-terminated pyrochlore bilayer, zr(zrce); and the supported Zr-terminated pyrochlore bilayer, zr(cezr). E_{PBE}: Adsorption energy calculated using the PBE functional. E_{DF2}: Adsorption energy calculated using the DF2 functional. $d_{O\text{-}H}$: Distance between one hydrogen and the nearest oxygen. $d_{Ce\text{-}H2}$: Distances between the two hydrogen atoms and the cerium atom below. $d_{H\text{-}H}$: Distance between the two hydrogens.

Model	zr2ce	zr2(ce)	zr2(zr)	zr3
Ce/Zr Surface Ratio	100/0	75/25	25/75	0/100
Surface Energy (oxidized) J/m^2	0.63	0.48	0.58	0.76
Surface Energy (reduced) J/m^2	1.30	0.86	0.48	0.76

Table S2.- Surface Energy (J/m^2) for the different models, related to Figures 5 and S10. Considered cases: the supported ceria monolayer, zr2ce; the supported Ce-rich pyrochlore monolayer, zr2(ce), the supported Zr-rich pyrochlore monolayer, zr2(zr), and the free zirconia relaxed surface, zr3. The data include the original models considering only Ce^{4+}, third row, or considering the cerium fully reduced to Ce^{3+}, by incorporating oxygen vancancies in the models, fourth row. The number of oxygen vacancies in the models are 8, 6, 2 and 0, respectively.

E (eV)	zr(zrce)	zr(cezr)
$E_f(2OH^-)$	-2.06	-2.40
$E_A(TS)$	1.14	0.85
$E_A(H^+-H^-)$	1.12	0.88
$E_f\,(H_2O)$	1.42	-0.91
$E_A\,(H_2O)$	1.42	0.02
$E_d(H_2O)$	-0.06	0.63

Table S3.- Energies (in eV) of the different elementary steps involved in the hydrogen molecule dissociation and the formation/release of the water molecule for the two supported pyrochlore bilayer models, related to Figures 5, 6 and 7. $E_f(2OH^-)$ corresponds to the enthalpy of the hydrogen dissociation process, where two hydroxyls groups are formed. $E_A(TS)$ is the activation barrier for this process, calculated from the transition state by the CI-NEB method. $E_A(H^+-H^-)$ corresponds to the activation barrier calculated using the frozen heterolytic model (values very close to the one calculated from the true transition state). $E_f(H_2O)$ corresponds to the formation energy of an adsorbed water molecule from two adjacent hydroxyls groups. $E_A(H_2O)$ is the energy barrier of this process, calculated using the CI-NEB method. $E_d(H_2O)$ corresponds to the water desorption energy.

Model	zr2ce	zr(zrce)	zr(cezr)	zr3
Ce/Zr Surface Ratio	100/0	75/25	25/75	0/100
Surface Energy (oxidized) J/m^2	0.63	0.67	1.06	0.76
Surface Energy (reduced) J/m^2	1.30	1.21	0.62	0.76

Table S4.- Surface Energy (J/m^2) for the different models, related to Figures 8 and S13. Considered cases: the supported ceria monolayer, zr2ce; the supported Ce-terminated pyrochlore bilayer, zr(zrce), the supported Zr-terminated pyrochlore bilayer, zr(cezr), and the free zirconia relaxed surface, zr3. The data include the original models considering only Ce^{4+}, third row, or considering the cerium fully reduced to Ce^{3+}, by incorporating oxygen vancancies in the models, fourth row.

E (eV)	O(Ce3)	O(ZrCe2)	O(Zr3)	O(CeZr2)
ce3	2.70	---	---	---
zr2ce	4.45 (3.40)	---	---	---
zr(zrce)	4.44	1.90	---	---
zr(cezr)	---	---	0.80	0.32
zr2(zr)	---	---	0.10	0.10

Table S5.- Surface oxygen vacancy formation energies (in eV) for the different models, related to Figures 8, S10 and S13. Considered cases: ce3 (pure ceria surface), zr2ce (supported ceria monolayer), zr(zrce) (supported Ce-rich terminated pyrochlore bilayer), zr(cezr) (supported Zr-rich terminated pyrochlore bilayer), and zr2(zr) (supported Zr-rich pyrochlore monolayer) for different configurations. Coordination of the original surface oxygen atom: O(Ce3), coordinated to 3 cerium atoms; O(Zr3), to 3 zirconium atoms; O(ZrCe2), to 1 zirconium and 2 cerium atoms; and O(CeZr2), to 1 cerium and 2 zirconium atoms. In the case of model zr2ce two possible configurations for the position of Ce^{3+} are considered.

2.- Figures

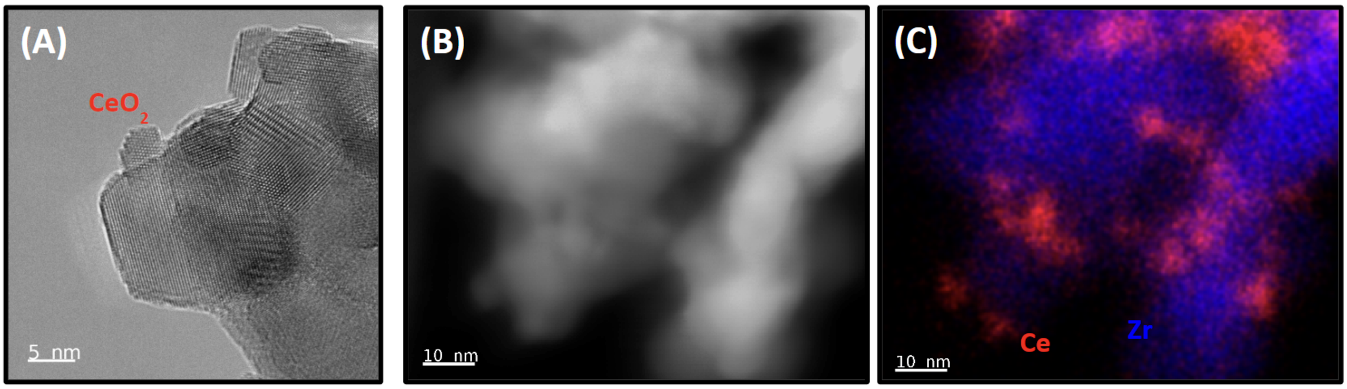

Figure S1.- TEM/STEM results corresponding to the fresh CZ1 sample, before any thermal treatment, related to Figure 1. (A) High resolution image evidencing the presence of 3D CeO_2 nanoparticles deposited onto the surface of YSZ crystals. (B) A representative HAADF-STEM image. (C) STEM-EDX elemental map corresponding to the HAADF image, showing the spatial distribution of Ce (in red) and Zr (in blue).

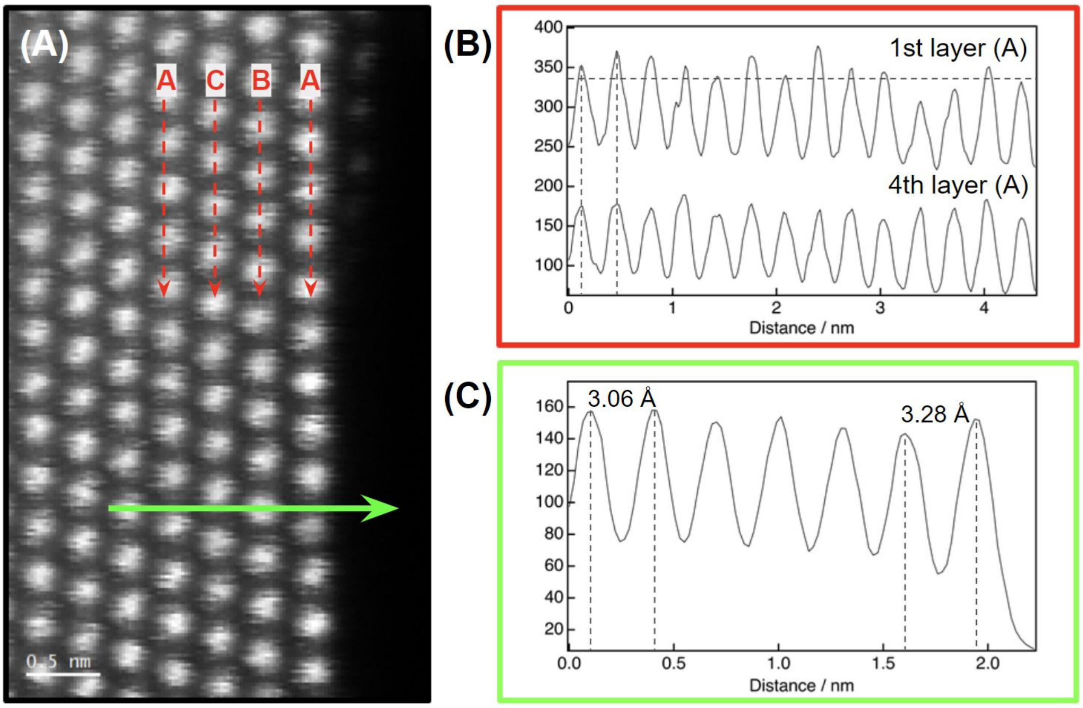

Figure S2.- HAADF-STEM details about the surface structure of CZ1 sample after a SO treatment, related to Figure 1. (A) Zoom on the surface corresponding to the nanostructure previously shown in the HAADF-STEM image of Figure 1(C). (B) Intensity profiles along the path marked with red arrows labeled as A. The direction perpendicular to the red arrows corresponds to the [111] zone axis, along which the fluorite cationic sub-lattice can be described as an ABC stacking of close-packed planes. Therefore, the 1st and 4th layers should lay at identical positions, allowing the maxima from both profiles to match, as confirmed by the vertical dashed line. This suggests a perfectly coherent growth of the ceria layer on top of YSZ (111) surface and, therefore, a contraction of the ceria lattice. In addition, maxima variations in the intensity profile recorded along the top-most surface layer (1st cationic plane), pointed by the horizontal dashed line, suggests that there is no cationic order within this layer. (C) The intensity profile along the [111] direction, green arrow, evidences an outward shift of the last layer, which corresponds to the Ce-rich fluorite-like monolayer, in good agreement with DFT calculations.

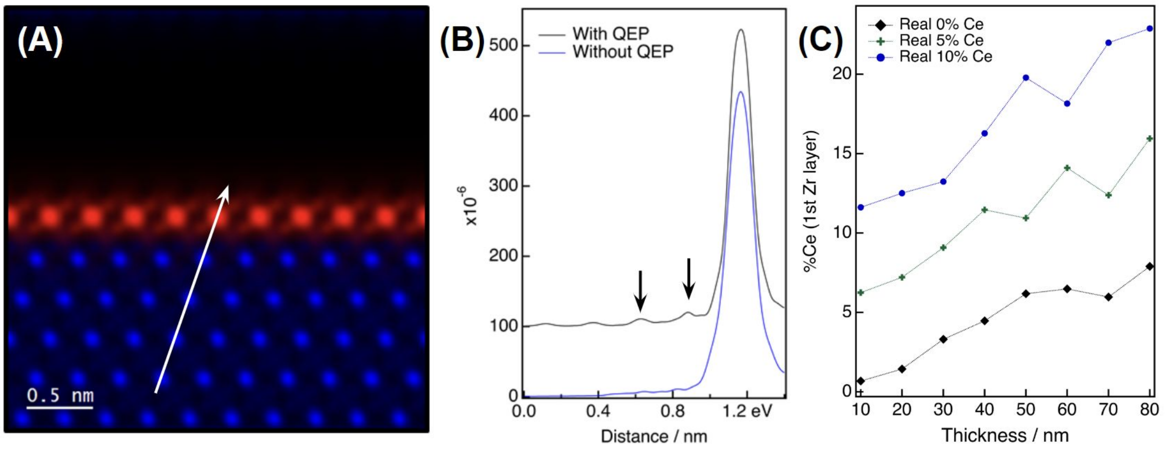

Figure S3.- Analysis of the influence of channeling/cross-talk artifacts on the quantification of Ce content in zirconia supported ceria monolayers, related to Figure 1. (A) XEDS simulation of a structural model which considers a CeO_2 monolayer grown onto a ZrO_2 crystal. Ce and Zr elemental distributions are shown in red and blue, respectively. The simulation was performed using the μSTEM code and considering the Quantum Excitation Phonons (QEP) model during calculation; (B) (black plot) Ce intensity profile along the path marked with a white arrow. (blue plot) the same profile without including QEP. The intense peak in both profiles corresponds to the top-most Ce layer. Note the presence of a weak, residual, Ce signal in the second and third layers when the calculation includes QEP, despite no Ce atoms are present in the model at those locations. For a 40 nm sample thickness, like that in the model, the apparent Ce content at the 1st Zr layer due to channeling/cross-talk propagation is about 4-5%. (C) Effect of model thickness onto the apparent concentration of Ce at the 1st Zr layer (black curve). Green and blue plots correspond to models in which a 5% and 10% actual Ce content is incorporated into the 1st Zr layer. Note how the apparent and actual Ce concentrations behave roughly as additive effects.

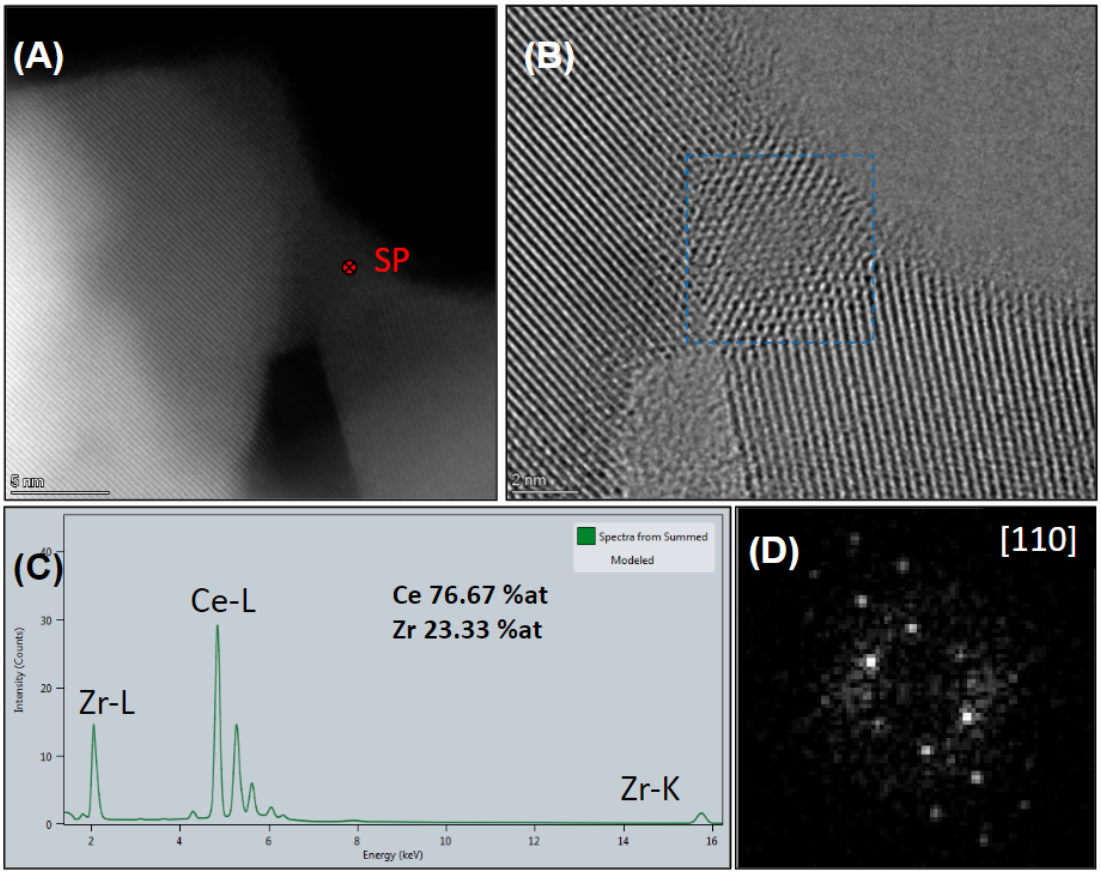

Figure S4.- TEM/STEM results corresponding to sample CZ1 after a SO-SRMO treatment, related to Figure 2. (A) HAADF image showing a Ce-rich nanocrystal. (B) HRTEM image of the same crystal. (C) The EDX analysis showing the Ce/Zr composition (D) Fourier components from the HRTEM image showing a flourite-like structure along [110] zone axis.

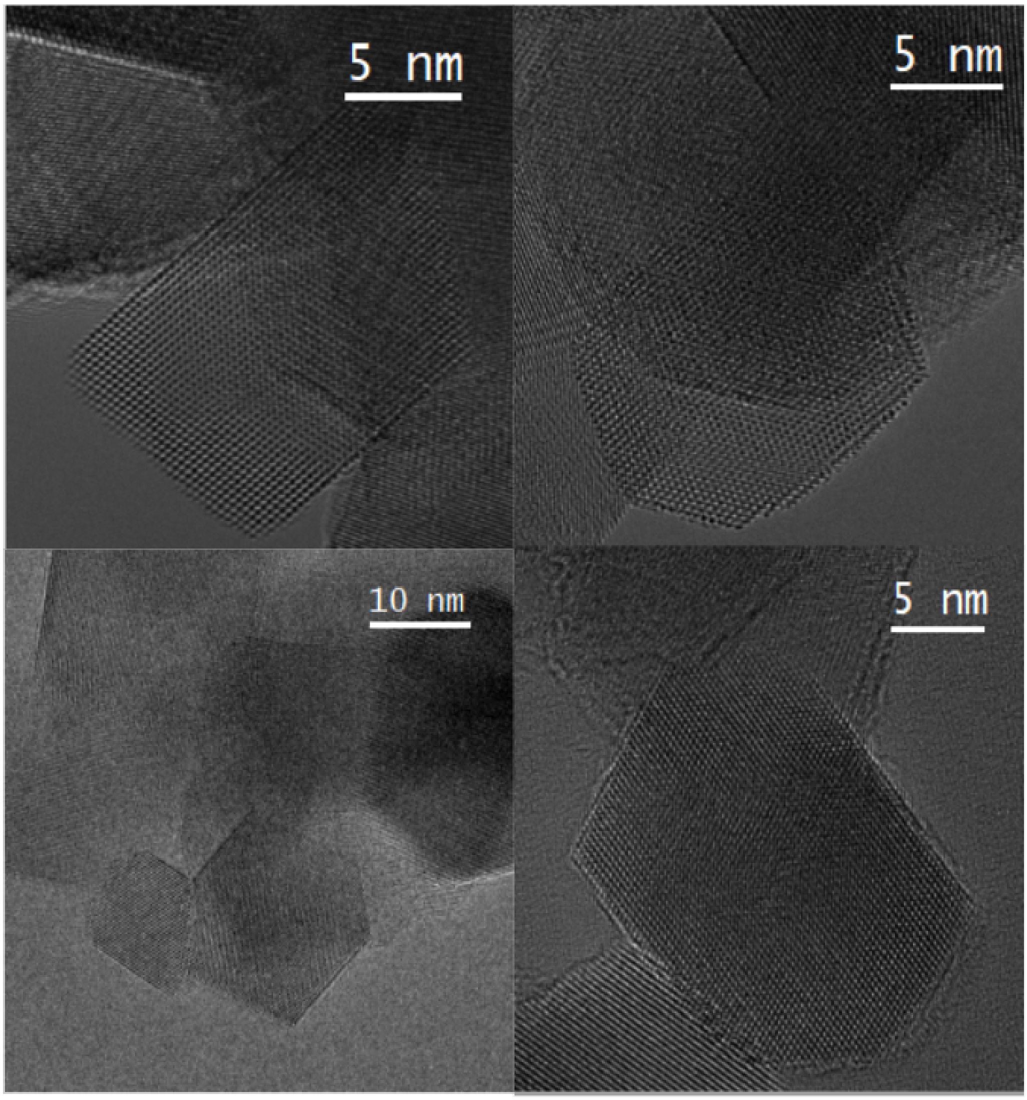

Figure S5.- Set of HRTEM images of the fresh CZ2 sample, related to Figure 3. They show the high dispersion of the cerium phase, and the high degree of exposure of (111) planes.

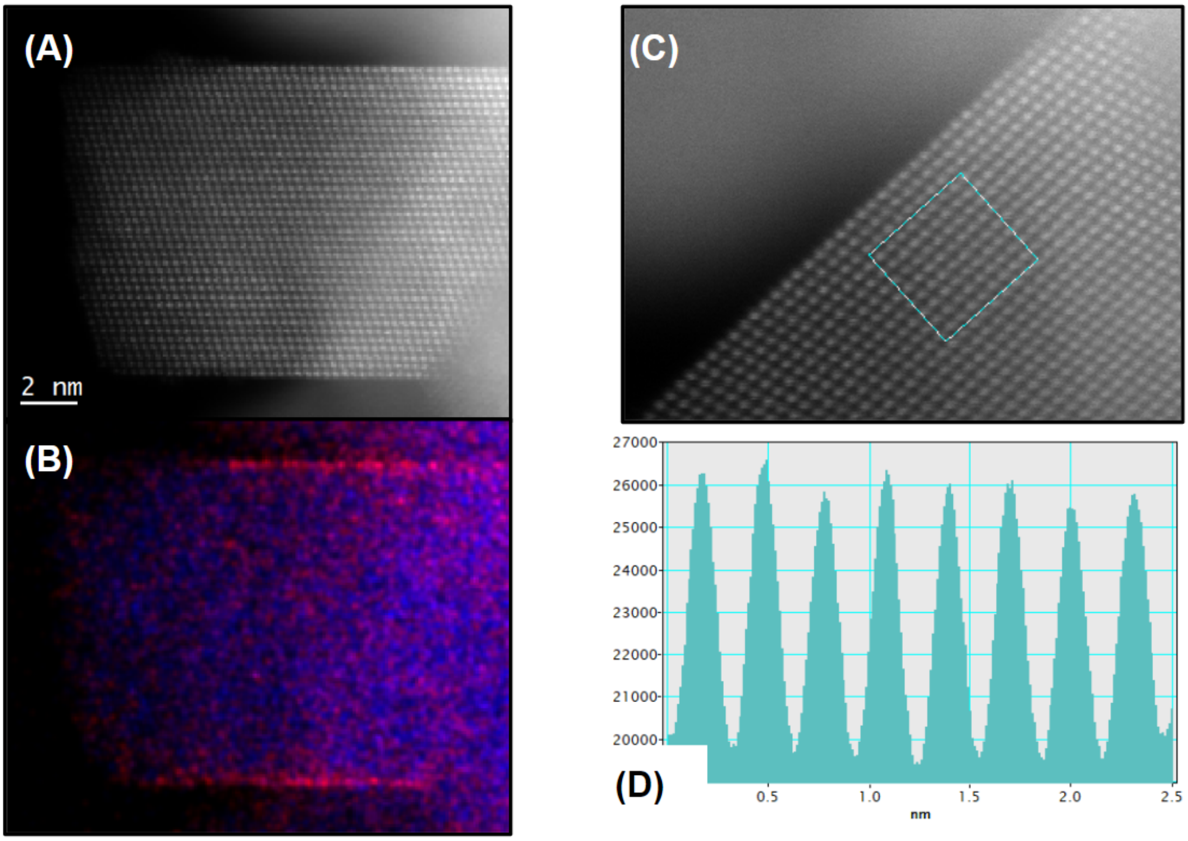

Figure S6.- STEM data of a small nanocrystal belonging to CZ2 sample after a SO-SRMO treatment, related to Figure 3. (A) Experimental, aberration-corrected, ADF-STEM image showing this nano crystal along the [110] zone axis. (B) The corresponding EDX map shows the presence of Ce atoms just on the surface, as expected. (C) Experimental atomic-resolution HAADF-STEM image close to the surface of the crystal. (D) Intensity profile of the selected area marked on the HAADF-STEM image showing the alternate intensity pattern of the {111} planes.

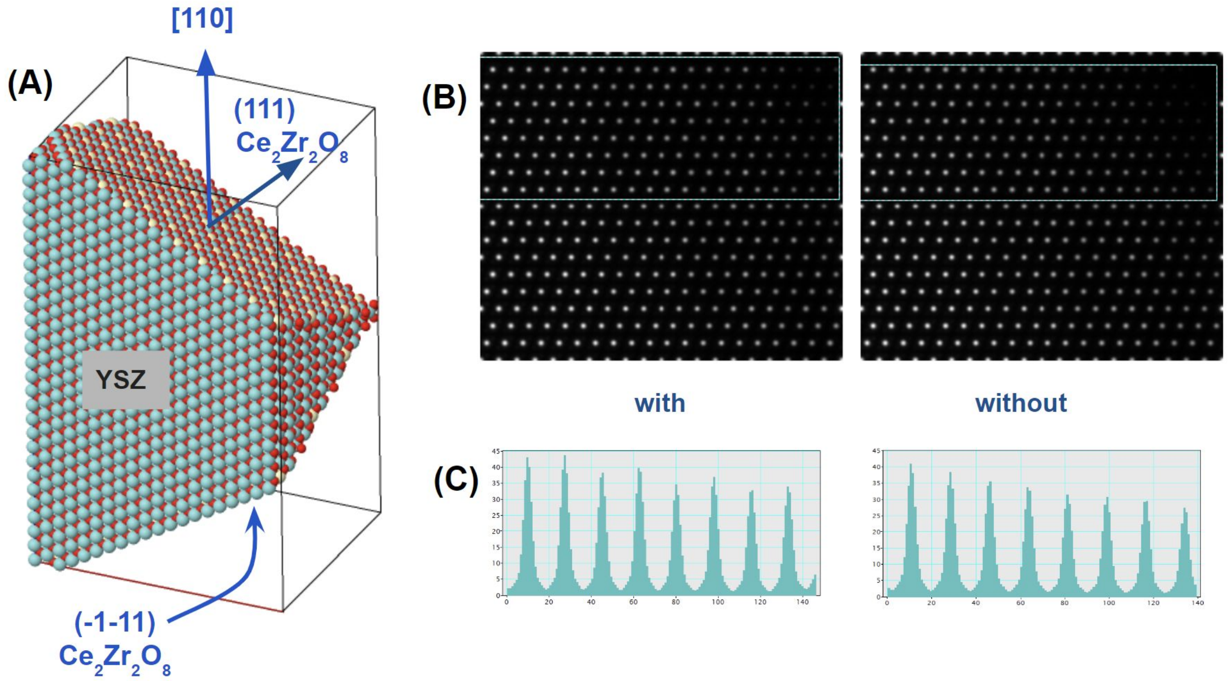

Figure S7.- HAADF-STEM image simulation of a small nanocrystal belonging to CZ2 sample after a SO-SRMO treatment, related to Figure 3. (A) Supercell containing a pure ZrO_2 crystallite viewed along the [110] direction (Z-axis of the supercell) bounded by (111) and (-1-11) facets. The thickness of the crystallite changes from 10 nm to almost zero, in good agreement with the experimental image in Figure S6. Two additional Zr-rich pyrochlore monolayers have been added to the model in order to simulate the effect of the supported ceria-based phase. The {111} planes are perpendicular to the Y-axis of the supercell. (B) HAADF-STEM image simulations (with and without the pyrochlore monolayers) calculated using TEM-SIM software and considering the following experimental conditions: High Voltage, 200 kV; Cs3, 0.001 mm; Cs5, 5 mm; defocus, 1.9 nm; objective aperture, 19 mrad; HAADF angles, 51-200 mrad; slice thickness, 0.181 nm. (C) Intensity profiles for the two simulated HAADF-STEM images showing that it is possible to detect the alternating intensities of the {111} planes with just one Zr-rich pyrochlore monolayer (¼ ceria coverage) grown onto ZrO_2 nanocrystals with thickness lower than 10 nm.

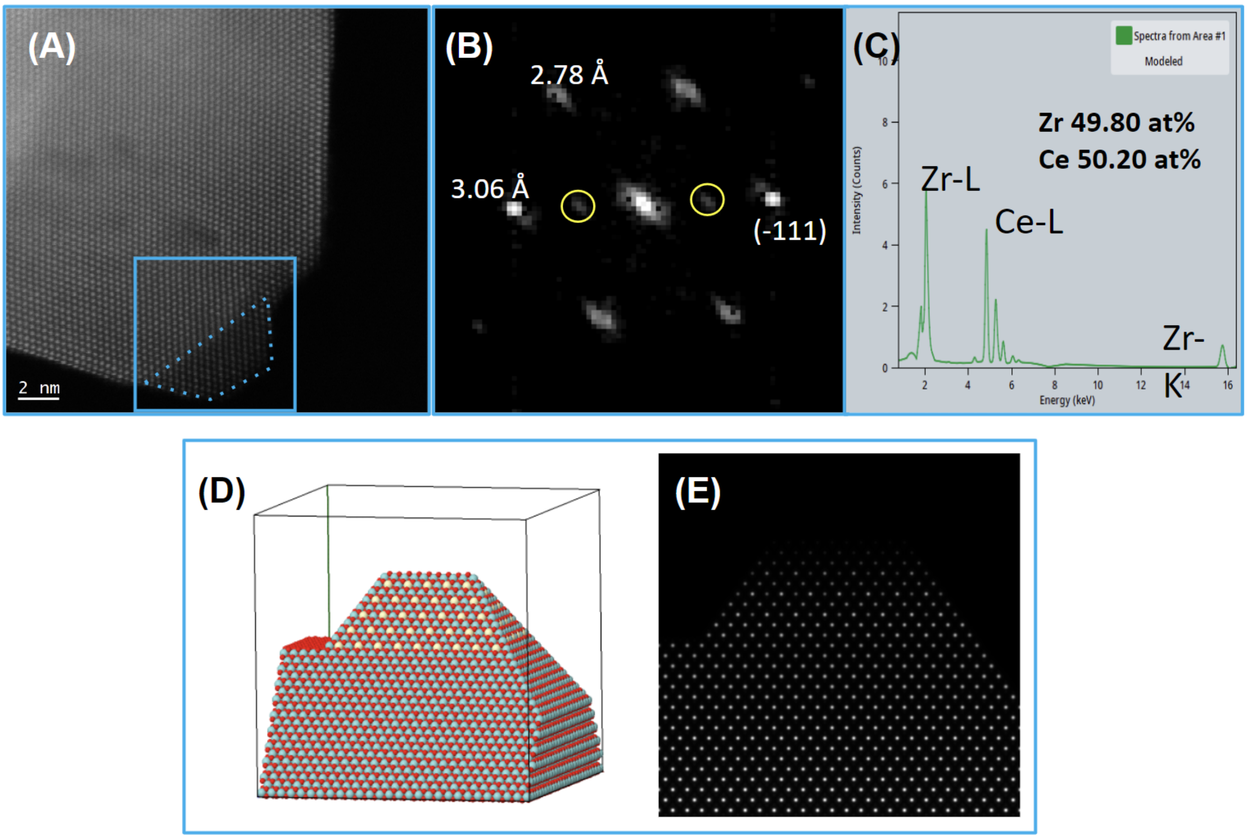

Figure S8.- STEM data of a supported ceria-based nanocrystal, found in CZ2 sample after a SO-SRMO treatment, related to Figure 3. (A) Aberration-corrected HAADF-STEM image of the CZ2 sample. In general, the phase containing ceria is extended along the (111) surfaces in the form of a 2D monolayer, but just a single accumulation was observed on a (001) surface as shown in this image. The contrasts of the atomic columns of the supported crystal denote a cationic ordering typical for a Ce/Zr pyrochlore structure. (B) Fourier Transform of the supported crystal showing the ½ (111) reflections characteristic of the pyrochlore structure. (C) The EDX analysis of the supported crystal reveals a composition close to 50/50 in good agreement with a bulk Ce/Zr mixed oxide. Note at this respect that the Ce/Zr composition of the Zr-rich pyrochlore monolayer is 25/75. (D) Complex model built using Rhodius software showing a Ce/Zr pyrochlore mixed oxide supported on a (001) plane of pure cubic YSZ crystal. Note the morphology of the nanocrystal exposing only (111) surfaces. A decrease in the intensity of the HAADF signal is expected in the Y-direction of the supercell, in good agreement with the experimental image. The pyrochlore monolayers have been omitted from support surfaces for clarity. (E) HAADF-STEM image simulation using TEM-SIM software and considering the following experimental conditions: High Voltage, 200 kV; Cs3, 0.001 mm; Cs5, 5 mm; defocus, 1.9 nm; objective aperture, 19 mrad; HAADF angles, 51-200 mrad.

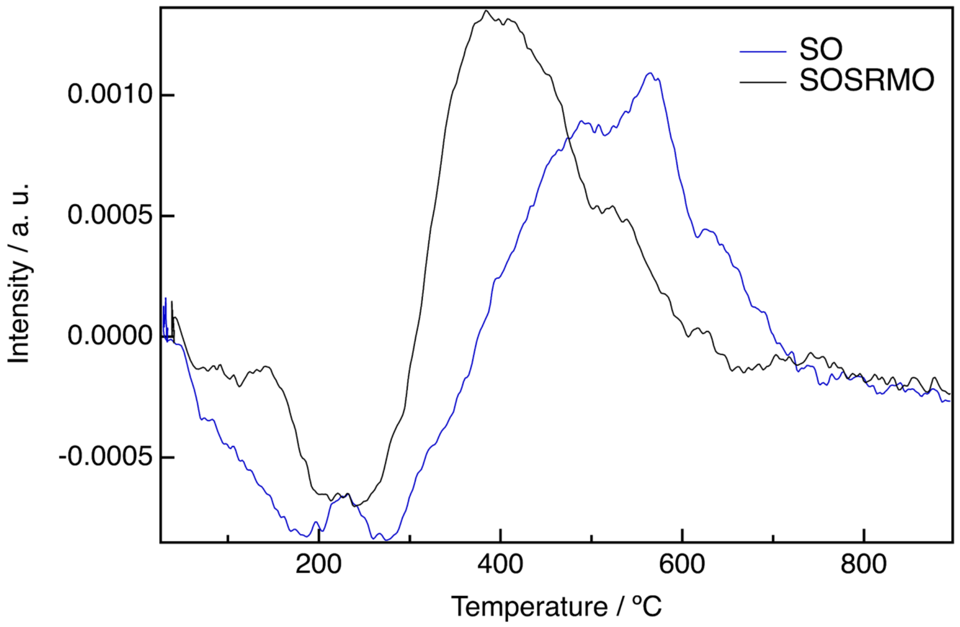

Figure S9.- Quantitative TCD-TPR experiments of the CZ2 sample after SO and SO-SRMO treatments, related to Figure 4. The hydrogen release signal at low temperature is due to desorption of hydrogen previously adsorbed at room temperature during the stabilization period of the experiment under hydrogen flow. Note that the release of hydrogen is delayed in the case of the SO-SRMO treatment, due to the stronger H_2 adsorption energy (see DFT calculations) characteristic of the pyrochlore monolayer with respect to pure ceria. This delay could be responsible for the displacement of the onset of the reduction of cerium, in the case of the SO-SRMO, from 220ºC to 300ºC with respect to the MS-TPR experiment shown in Figure 4. The consumption of hydrogen, 0.094 H mmol/g, calculated by integration of the corresponding curve area, is almost 100% equivalent to the amount of Ce phase for the SO sample (considering one Ce^{3+} for one atomic H). In the case of the SO-SRMO is 95%, 0.087 H mmol/g. The difference could be attributed again to the delay in the release of molecular hydrogen. In conclusion, both TPR experiments show that the consumption of hydrogen is due to the reduction of the Ce-containing phase and the improvement of the reducibility of the sample at low temperature in the case of SO-SRMO sample.

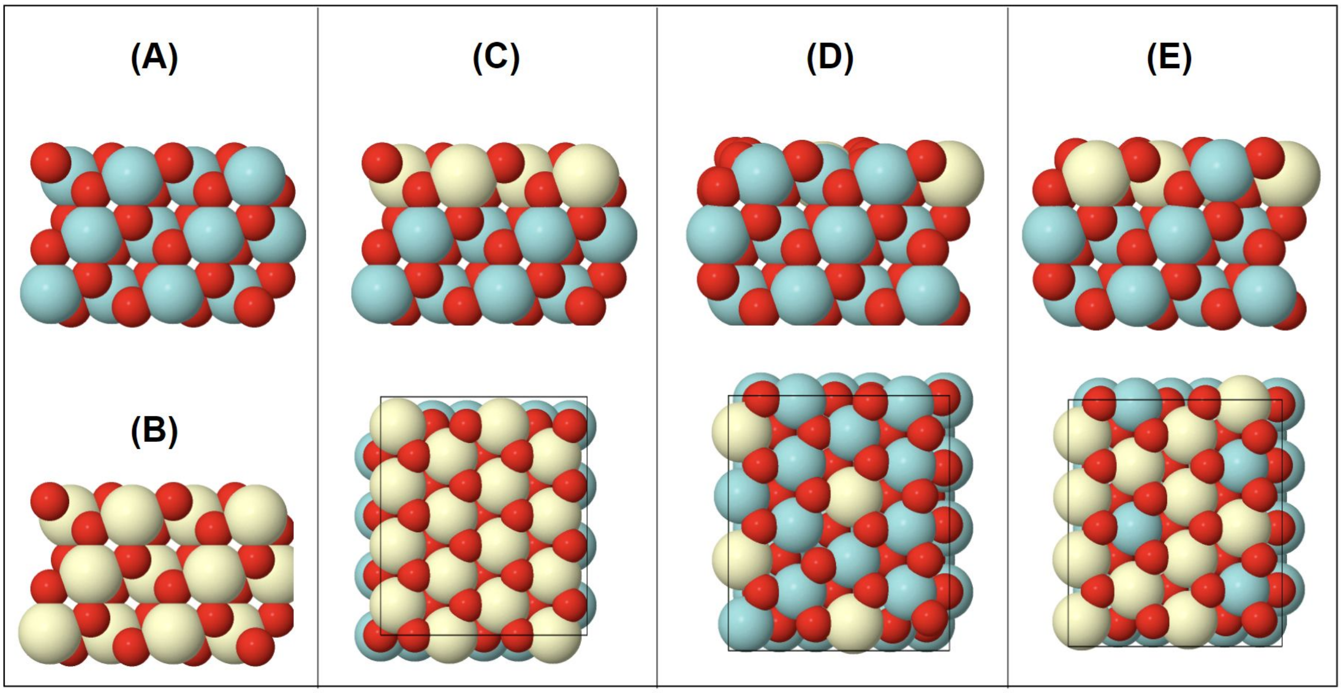

Figure S10.- Set of supercells for the surface calculations, related to Figure 5 and Table 1. All of them correspond to a fluorite-like three (111) layers structure. Each layer is made of the 3 atomic planes: O-M-O. The bottom layer is kept fixed while the other two are allowed to rearrange during the calculations. The models correspond to: (A) pure zirconia (111) surface, ce3 model, (B) pure ceria (111) surface, ce3 model, (C) zirconia supported ceria (111) monolayer, zr2ce model, (D) zirconia supported Zr-rich pyrochlore (111) monolayer, zr2(zr) model, and (E) zirconia supported Ce-rich pyrochlore (111) monolayer, zr2(ce) model. All the models contain 144 atoms. The dimensions of the models (12.52 Å x 14.45 Å x 22.00 Å) are adjusted for the lattice parameter of a DFT relaxed ZrO_2 (a=5.11 Å) structure, with the exception of model b, whose dimensions (13.24 Å x 15.50 Å x 22.00 Å) were adjusted for the lattice parameter of a DFT relaxed CeO_2 (a=5.48 Å) structure. Note the unique structure of the Zr-rich monolayer (D) consisting of a compact arrangement of isolated cerium atoms resembling a single-atom catalyst.

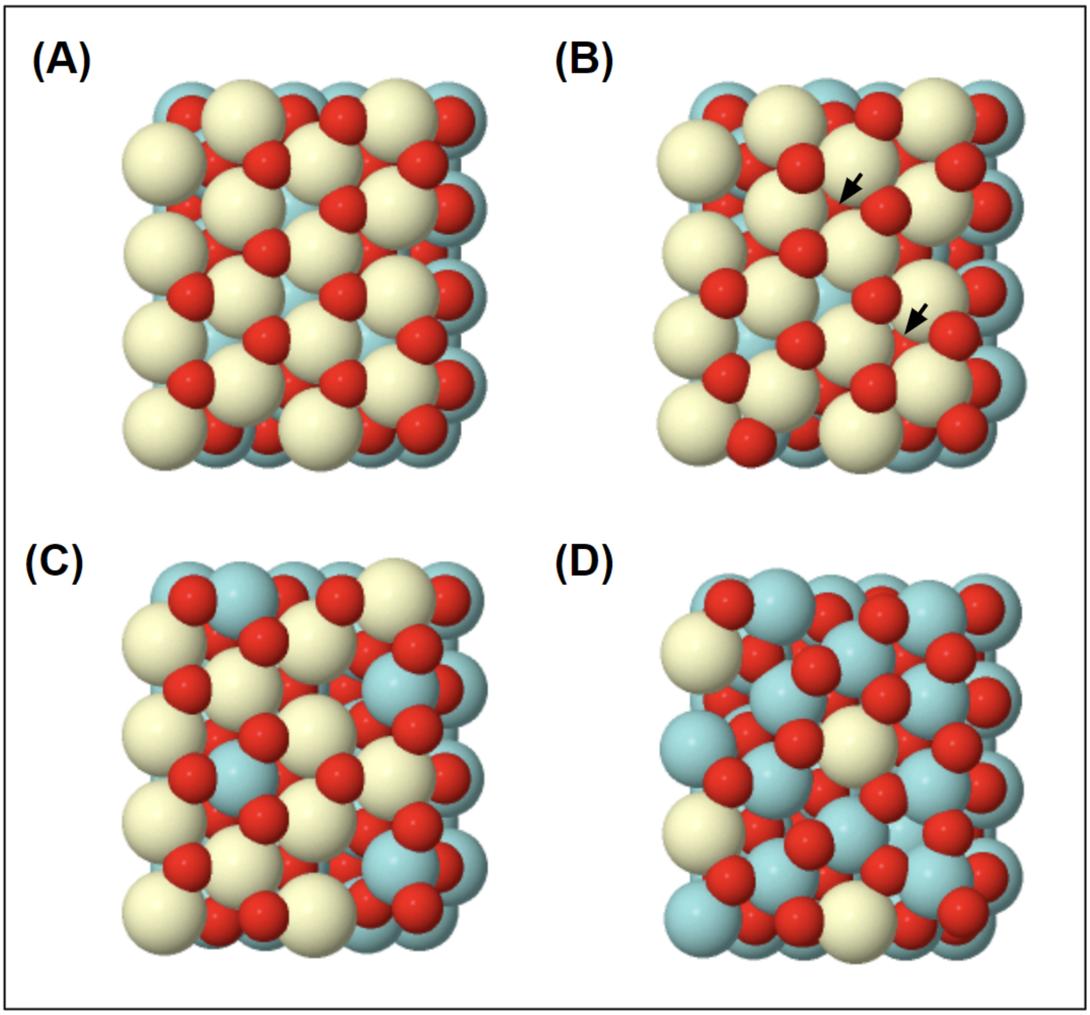

Figure S11.- Models corresponding to the supported reduced monolayer phases, related to Figure 5. (A) Unrelaxed C-Ce_2O_3 monolayer epitaxially grown on top of a ZrO_2 (111) surface. (B) The same model after relaxation. It is possible to observe that two oxygen atoms from the fourth atomic plane move to the third atomic plane during the relaxation process, as depicted with black arrows. The oxygen vacancies located this way in the fourth atomic plane allow the reduction of the coordination number of the Zr atoms of the support. However, the number of surface oxygen vacancies remained unchanged. (C) The reduced Ce-rich pyrochlore monolayer after relaxation. The model contains 12 Ce atoms and 6 O vacancies. Testing several configurations, the most stable one is obtained considering 4 oxygen vacancies in the fourth atomic plane (reducing again the coordination number of the Zr atoms of the support) and the remaining two on the surface. (D) The reduced Zr-rich pyrochlore monolayer after relaxation. The model contains 4 Ce atoms and two O vacancies located this time one on the fourth atomic plane and one on the surface.

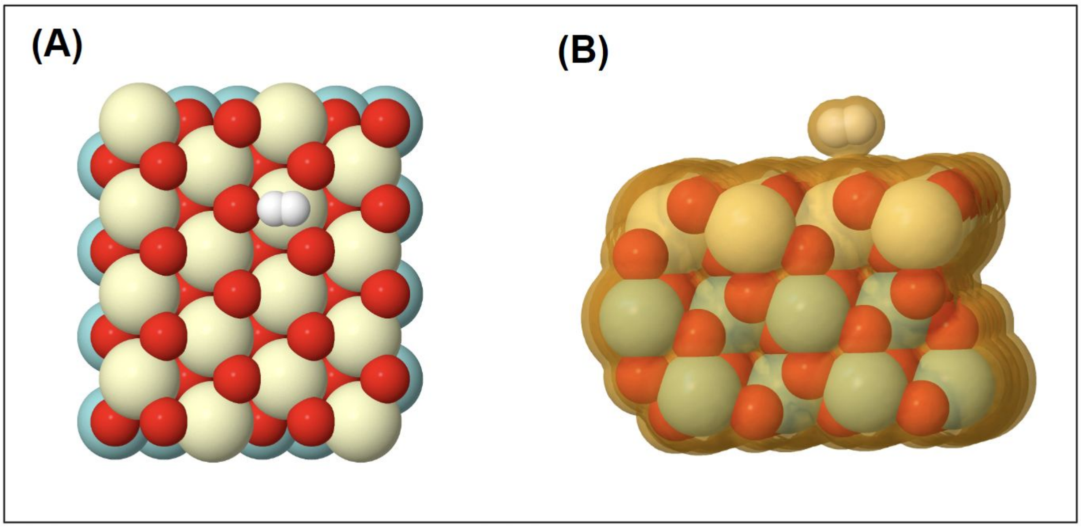

Figure S12.- Hydrogen molecule adsorbed on a supported ceria (111) monolayer, related to Figure 6. (A) Top view of the model (B) Profile view of the model showing the Van der Waals surface. The relaxed supercells for the rest of the models are very similar.

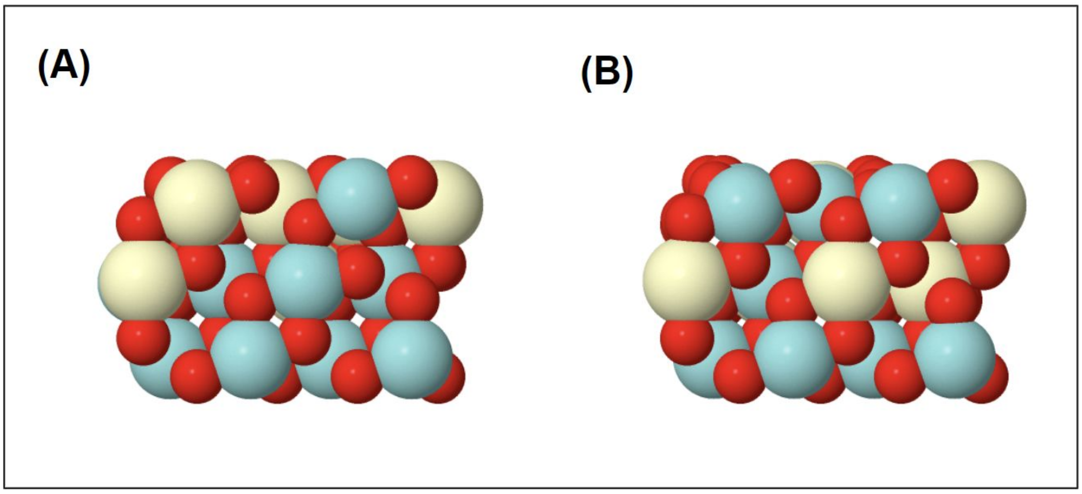

Figure S13.- Zirconia supported pyrochlore (111) bilayer models, related to Figure 8. (A) Supported Ce-rich terminated pyrochlore (111) bilayer, zr(zrce) model, (B) Supported Zr-rich terminated pyrochlore (111) bilayer, zr(cezr) model. Both models contain 144 atoms. The dimensions of the models (12.52 Å x 14.45 Å x 22.00 Å) are adjusted for the lattice parameter of a DFT relaxed ZrO_2 (a=5.11 Å) structure.

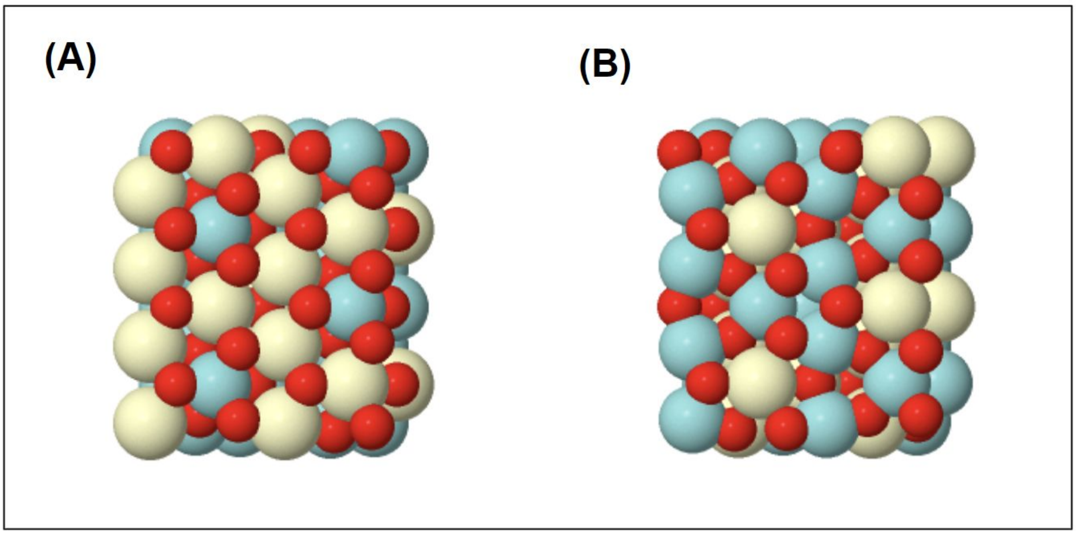

Figure S14.- Models corresponding to the supported reduced bilayer phases, related to Figure 8. (A) The reduced Ce-terminated pyrochlore bilayer model. O vacancies are located in the fourth and sixth planes, in the Zr-rich layer, as it happens in the bulk $Ce_2Zr_2O_7$ phase. (B) The reduced Zr-terminated pyrochlore bilayer model. O vacancies located at the first and third atomic planes, in the Zr-rich layer, as it happens in the bulk $Ce_2Zr_2O_7$ phase. In both cases, the oxygen vacancies don't move during the relaxation process.

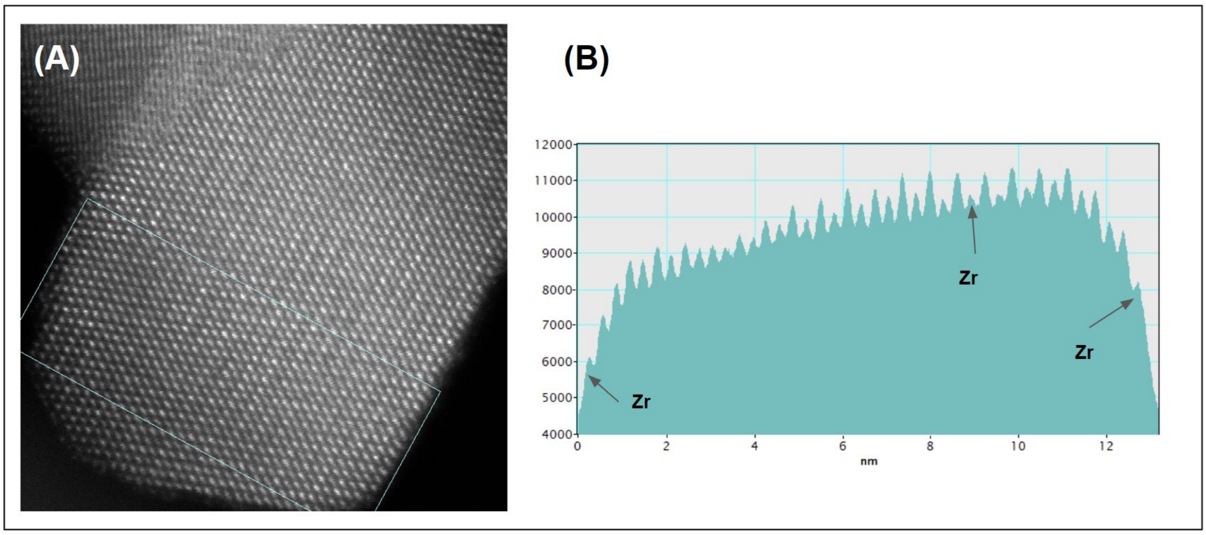

Figure S15.- STEM data corresponding to a Ce/Zr mixed oxide (composition 50/50) after a SRMO treatment, related to Figure 8. (A) Aberration-corrected STEM-HAADF image showing the typical pyrochlore-like structure. (B) Intensity profile showing the alternating Zr-rich and Ce-rich (111) planes. Note the Zr-terminated surfaces.
